# Reduction of Ca_v_1.3 channels in dorsal hippocampus impairs the development of dentate gyrus newborn neurons and hippocampal-dependent memory tasks

**DOI:** 10.1371/journal.pone.0181138

**Published:** 2017-07-17

**Authors:** Su-Hyun Kim, Ye-Ryoung Park, Boyoung Lee, Byungil Choi, Hyun Kim, Chong-Hyun Kim

**Affiliations:** 1 Center for Neuroscience, Korea Institute of Science and Technology, Seoul, Korea; 2 Neuroscience Program, Division of Bio-Medical Science and Technology, KIST School, Korea University of Science and Technology, Seoul, Korea; 3 Center for Cognition and Sociality, Institute for Basic Science, Daejeon, Korea; 4 Department of Anatomy and Division of Brain Korea 21 Biomedical Science, College of Medicine, Korea University, Seoul, Korea; University Paris Diderot, FRANCE

## Abstract

Ca_v_1.3 has been suggested to mediate hippocampal neurogenesis of adult mice and contribute to hippocampal-dependent learning and memory processes. However, the mechanism of Ca_v_1.3 contribution in these processes is unclear. Here, roles of Ca_v_1.3 of mouse dorsal hippocampus during newborn cell development were examined. We find that knock-out (KO) of Ca_v_1.3 resulted in the reduction of survival of newborn neurons at 28 days old after mitosis. The retroviral eGFP expression showed that both dendritic complexity and the number and length of mossy fiber bouton (MFB) filopodia of newborn neurons at ≥ 14 days old were significantly reduced in KO mice. Both contextual fear conditioning (CFC) and object-location recognition tasks were impaired in recent (1 day) memory test while passive avoidance task was impaired only in remote (≥ 20 days) memory in KO mice. Results using adeno-associated virus (AAV)-mediated Ca_v_1.3 knock-down (KD) or retrovirus-mediated KD in dorsal hippocampal DG area showed that the recent memory of CFC was impaired in both KD mice but the remote memory was impaired only in AAV KD mice, suggesting that Ca_v_1.3 of mature neurons play important roles in both recent and remote CFC memory while Ca_v_1.3 in newborn neurons is selectively involved in the recent CFC memory process. Meanwhile, AAV KD of Ca_v_1.3 in ventral hippocampal area has no effect on the recent CFC memory. In conclusion, the results suggest that Ca_v_1.3 in newborn neurons of dorsal hippocampus is involved in the survival of newborn neurons while mediating developments of dendritic and axonal processes of newborn cells and plays a role in the memory process differentially depending on the stage of maturation and the type of learning task.

## Introduction

L-type calcium channels (LTCCs) are formed by the Ca_v_1 family, which comprise isoforms of Ca_v_1.1–4 [1]. In neurons, both Ca_v_1.2 and Ca_v_1.3 are expressed [2] and are known to regulate neuronal excitability [3, 4], gene expression [5, 6], synaptic plasticity [3, 7–9], and learning and memory [10]. Pharmacological agents such as dihydropyridine derivatives have been used as blockers to find roles of LTCCs in hippocampal-dependent learning and memory [11–13]. However, it has been difficult to define functions of specific isoforms of LTCCs due to the non-specific sensitivity of blockers to isoforms of LTCCs and their toxicity [14]. Recently, genetic methods were used to investigate functions of each isoform in hippocampal-dependent learning and memory [15–20]. Ca_v_1.2 conditional KO (cKO) mice, where Ca_v_1.2 was deleted in the forebrain area including hippocampus and cortex, showed that consolidation of memory, ≥ 3 days old, of the Morris water-maze learning was defected [15, 17]. Null Ca_v_1.3 KO mice showed impairment in the recent memory of object location recognition (OLR) task [21] and CFC though there were no effects in extinctions at 2 to 3 days after training and in the recent memory of water-maze learning [16]. These genetic studies suggest differential roles of Ca_v_1.2 and Ca_v_1.3 even in hippocampal-dependent learning and memory tasks. Moreover, it is still unclear how hippocampal Ca_v_1.3 contributes to CFC learning and memory and whether the remote memory of CFC is affected or not in Ca_v_1.3 KO mice.

Adult hippocampal neurogenesis occurs in DG subgranular zone and has been suggested to be involved in the acquisition of new learning and memory processes [22, 23]. Ablation of adult hippocampal neurogenesis using x-ray irradiation or pharmacological methods has impaired hippocampal-dependent memories [24–28]. CFC memory is hippocampus-dependent [29] and survival of adult newborn neurons is shown important in single-trial CFC learning and memory [27, 28]. However, it is largely unknown what cellular and molecular mechanism could link the survival of adult newborn neurons with learning and memory processes. It has been shown that activity-dependent regulation of neurogenesis might be related with LTCCs [30–33]. Recently, reduction of survival of adult newborn neurons in adult hippocampus was observed in Ca_v_1.3 KO mice and forebrain-specific Ca_v_1.2 cKO mice [20, 34]. These studies suggest an idea that LTCCs can affect hippocampal-dependent learning and memory processes via their role in adult neurogenesis. Therefore, it will be interesting to know what endogenous functions of Ca_v_1.3 have in the development of adult newborn neurons, which can be critical for learning and memory.

In this study we aimed to determine what developmental stages of adult hippocampal neurogenesis are directly affected by Ca_v_1.3, how Ca_v_1.3 regulates the development of adult newborn neurons, and in what region, such as dorsal vs ventral, of hippocampus Ca_v_1.3 is important for CFC learning. To achieve these aims, hippocampal neurogenesis was quantified along a month period of time and the morphology of dendrites and axon terminals of DG newborn neurons of Ca_v_1.3 KO mice was investigated. To confirm the effect of hippocampal-dependent memory tasks in KO mice, effects of AAV- or retrovirus-mediated Ca_v_1.3 KD of dorsal hippocampal area on learning and memory tasks were examined. The results showed that Ca_v_1.3 in dorsal hippocampal newborn neurons affects the survival and development of newborn neurons and is involved in the recent CFC memory, and Ca_v_1.3 in mature neurons seems to contribute to both recent and remote memory of CFC learning.

## Materials and methods

### Animals

The animal procedures were in accordance with the guidance of the principles in the care and use of experimental animals which were set by the Animal Care and Use Committee of Korea Institute of Science & Technology (ACUCK) and all animal experiments were approved by ACUCK done in this study where male 8 to 12 week old mice were used. In the Ca_v_1.3 KO mice, the gene for the pore-forming subunit of the Ca_v_1.3 calcium channel has been deleted by insertion of a neomycin cassette into exon 2, which results in a complete null mutation [35]. Mice were maintained in two genetic backgrounds, either 129/sv or C57BL/6J. KO and WT littermate mice were generated by mating heterozygotes from two genetic backgrounds (129/sv and C57BL/6J). All animals were kept at 23 ~ 25°C under light/dark (12:12 hour) cycle and given *ad libitum* access to food and water.

### Production of AAV and retrovirus

Candidate shRNAs targeting Ca_v_1.3 were, 24 base pair sequence long, designed by RNAi design program (Integrated DNA Technologies). A loop sequence (CTTCCTGTCA) was inserted between antisense (TTATCTCTCATGGCAACTTTCCCA) and sense (TGGGAAAGTTGCCATGAGAGATAAA) sequences. DNA oligomers were synthesized and cloned into a modified pAAV-MCS vector, pAAV-shRNA (provided by Dr. Ralph J. DiLeone, Yale University School of Medicine). The insertion was confirmed by sequencing and the best candidate was selected by measuring KD efficiency with quantitative real-time polymerase chain reaction (qRT-PCR) after transfecting candidate plasmids into primary neuronal culture. KD control GFP-AAV carries a scrambled shRNA sequence. To produce high titer AAV (1 x 10^9^ ~ 10^11^ pfu/ml), the target plasmid, pRC and pHelper plasmids (gift from Dr. R. J. Dileone), were transiently transfected to HEK293TN cells. Cell lysates harvested at 72 hours after transfection were treated with benzonase (50 unit/ml; Sigma, USA) and virus particles were purified and concentrated with heparin column (GE healthcare, Sweden) and 100k filtering tube (Millipore, USA). Virus titers were determined by counting GFP (+) HEK293T cells at 48 hours after infection. Retroviral vector (CAG-GFP, gift from Dr. Fred H. Gage, Salk Institute, USA) was used for making retrovirus to label adult newborn neurons of DG area of mouse [36]. To KD Ca_v_1.3, Ca_v_1.3 targeting shRNA candidates, 19 base pair sequence long, were designed by RNAi design program (IDTdna, USA). A loop sequence (TTCAAGAGA) was inserted into between sense (GGCCCGCGTTGCTGTACAA) and antisense (TTGTACAGCAACGCGGGCC) sequences.

DNA oligomers were synthesized and cloned into the retrovirus plasmid (RNAi-Ready pSIREN-RetroQ vector, Clontech, Japan). The insertion was confirmed by sequencing and KD efficiency was measured by qRT-PCR at 48 hours after transfection into HT22 cell line. KD control GFP-retrovirus carries a scrambled shRNA sequence. To change or enhance the fluorescence of retrovirus, Ca_v_1.3 targeting shRNAs were subcloned into pSUbGW plasmids (gift form Dr. H. Song, Johns Hopkins University, USA). To produce high titer retrovirus, retroviral vector and VSVG (gift from Dr. F. H. Gage) were transiently transfected to HEK293-based packaging cell line (Platinum-GP Retroviral Packaging Cell Line, Cell Biolabs, USA) and media were changed and collected at every 24 hour interval for 3 days after transfection (S6C and S6D Fig).

### Quantitative real-time polymerase chain reaction

Total cellular RNA was isolated from cells using the total RNA extraction kit or manually using trizol (Geneall, Korea). qRT-PCR was done using a real time PCR system (Applied Biosystems: 1 cycle at 50°C for 2 min & 95°C for 10 min; 40 cycles at 95°C for 15 s & 60°C for 1 min). Glyceraldehyde 3-phosphate dehydrogenase was used as a control for cDNA loading and PCR. PCR primers were synthesized by M-biotech Inc. (Korea). Ca_v_1.3 PCR primers were targeted to exon 21–22 (# Mm.PT.47.16004990).

### Stereotaxic viral injections

AAV (2 μl) or retrovirus (1.5 μl) containing solution was injected into the molecular layer of the DG (Dorsal hippocampus, anterior-posterior (AP): -2.0 mm, medial lateral (ML): ±1.5 mm, dorsal ventral (DV): -1.85 mm; Ventral hippocampus, AP: -2.8 mm, ML: ±3 mm, DV: -4 mm) using microsyringe pump (Micro 4, WPI, USA) and a calibrated 50 μl Hamilton syringe (Hamilton co., USA) fitted with a 33-gauge needle (WPI, USA) (0.1 μl/min). Mice were anesthetized with a mixture of avertin (200 mg/kg; Sigma, USA) and placed in a stereotaxic frame (Stoelting Co, USA). The final titers of retrovirus and AAV were ~10^8^ pfu/ml and 10^9^ ~ 10^11^ pfu/ml, respectively.

### Immunohistochemistry (IHC)

Bromodeoxyuridine (BrdU, Sigma, USA) was used to quantify the proliferation and survival of adult newborn neurons of DG in hippocampus. To quantify the proliferation rate of newborn neurons, mice were injected with BrdU (300 mg/kg in saline) once intraperitoneally and sacrificed at 24 hours after the BrdU injection. To quantify the survival rate of newborn neurons, mice were injected with BrdU (300 mg/kg in saline) once a day for 4 days intraperitoneally and sacrificed at 14 or 28 days after the last BrdU injection. For perfusion of brain, mice were anesthetized with avertin (200 mg/kg, Sigma) and transcardially perfused with cold 0.1M phosphate buffer saline (PBS) and then 10% neutral buffer formalin (NBF, Sigma). Brains were post-fixed overnight in 10% NBF at 4°C, then cryoprotected in 30% sucrose in PBS at 4°C for 2 days. Coronal section of 40 μm thick was cut using cryostat (HM525, Thermo scientific, USA). For immunostaining of BrdU, brain sections were pretreated 2 N HCl for 1 hour at 37°C and rinsed in 0.1 M borate buffer (pH 8.5) for 10 min. To block non-specific bindings, sections were incubated in 2% normal goat serum with 0.3% triton X-100 in PBS (Blocking solution) for 1 hour at room temperature (RT) and then incubated in rat anti-BrdU antibody (1:400, Serotec, USA) and mouse anti-NeuN antibody (1:400, Millipore, USA) in blocking solution for 16 hours at 4°C. After incubation, sections were washed in PBS and incubated in goat anti-rat 488 (1:400, life technology, USA) and goat anti-mouse 568 (1:400, Life technology, USA) antibodies in blocking solution for 2 hours at RT. For immunostaining of Ca_v_1.3 in mouse brain, animals were perfused with cold PBS and post-fixed with 10% NBF for 1 hour at RT. Sections were washed 3 times, each 10 min in 0.1 M PBS and then incubated in 4% normal goat serum (NGS, Vector laboratories, USA) in PBS containing 0.25% triton-X100 for 2 hours at RT. Then sections were incubated in rabbit anti-Ca_v_1.3 (1:200, Alomone Lab, Israel) in blocking solution for 72 hours at 4°C, washed in PBS and incubated in secondary antibodies in PBS containing 0.25% trition-X100 for 2 hours at RT.

### Image acquisition and analysis

Images were acquired using a confocal microscope (Fluoview 1000, Olympus, Japan). Retrovirus expressing eGFP was used to label adult newborn neurons [36]. Images of GFP (+) cells were acquired at 14 or 28 days after viral injection into dorsal hippocampal region. Images (40x objective lens), taken in 1 μm step, were used for detecting BrdU (+) cells and for analysis of morphology of dendrites of newborn neurons. Images (40x/6x-zoom) in 1 μm step were used to analyze the morphology of axon terminals. Images (60x/6x-zoom) were used to analyze the spine morphology and density. Fiji software (Image J, NIH, USA) was used to measure, reconstruct and analyze the length, complexity, branching points of dendrites and axon terminal morphology. NeuronStudio software (http://www.mssm.edu/cnic) was used to analyze the dendritic spine morphology and density of GFP (+) neurons [37]. The types of spine were classified as stubby, thin or mushroom by using the default values of the software (Neck Ratio, 1.1; Thin Ratio, 2.5; Mushroom size, 0.35 μm).

### Animal behavior experiments

#### Contextual fear conditioning (CFC)

The procedure for testing CFC memory was in accordance with the method of McKinney et al. (2006) [16]. Fear conditioning was carried out in the chamber (18 x 17.5 x 38 cm) (Med Associates, USA) containing a stainless-steel bar-grid floor (5 mm ϕ rods, spaced 1 cm apart). Electric shock was delivered through the bar-grid floor of the box connected to a programmable shocker. A light bulb and a fan were located inside the chamber. Every mouse was handled for 3 min per day for 5 days before the training session of CFC. On the 1^st^ training day (Day 0), mouse was given with a single shock (0.5 mA, 2 s) at 180 s after exposure to the chamber and then was returned to the home cage 30 s after the shock. Intensity of light bulb illumination inside chamber was 30 ~ 50 lux. At 24 hours after the 1^st^ training, the 2^nd^ training procedure was given while video recording the freezing behavior (Day 1). On the 3^rd^ day, mouse was placed in the same chamber for CFC memory test (Day 2). To assess the remote memory of CFC, freezing behavior was monitored for 3 min at 23 days after the 1^st^ training day.

#### Passive avoidance (PA)

The PA procedure of PA was described in Pan et al. (2012) [38]. In brief, on the training day (Day 0) mouse was placed in the lighted compartment, facing away from the dark compartment and the guillotine door was lifted open after 30 s free exploration. When mouse entered the dark compartment along with all four paws, the guillotine door was closed and the latency to enter was recorded (from the time when the door was lifted). A foot-shock was delivered (0.7 mA, 2 s) at 3 s after closing the door and 30 s later, the mouse was moved to the home cage. To measure the recent memory of PA learning, at 24 hours after the training, the mouse was returned to the lighted compartment, facing away from the dark compartment. After 30 s later, the guillotine door was open. Latency to enter the dark compartment was measured (Day 1). To measure the remote memory of PA, the latency was recorded at 21 and 42 days after the training day. Measurements at day 42 used mice only that did show the shock memory at day 21.

#### Object recognition (OR) and OLR tasks

Procedures of OR and OLR were based on Goodman et al. (2010) [21]. OR task was performed in an open field box (40 x 40 x 40 cm). Two types of objects were different in shape, color and texture. One of them was a yellow regular tetrahedron, made of acryl. The other one was a black & red color sphere, made of urethane. Both are 7 ~ 7.5 cm high. The objects were fixed to the ground of the box, not to be moved by mice. Sniffing objects was considered as the explorative action of mouse. OR test was composed of 3 steps such as habituation, training and test, and given once per day. During the habituation step, mouse was placed in an open field box for 30 min without objects. Then, during training period, two identical objects were presented to the mouse for 20 min. At 24 hours after the training, one of the familiar objects was replaced with a novel object and presented to the mouse for 10 min for the test. Procedures for OLR task were similar to OR task except that one of the objects was moved to a different location for the test.

### Statistics

Statistical values are presented as means ± S.E. and two-tailed unpaired t-test with α = 0.05 was used to compare data between two experimental groups unless otherwise mentioned. One-way or two-way ANOVA was applied to most analysis if applicable and post hoc (Bonferroni or Dunnett) analysis was followed (SPSS v.24, IBM, USA). G = genotype, T = time or trials, D = distance, S = Sound dB. The results of ANOVA and post hoc analysis were provided in Supporting Information.

## Results

### Expression of Ca_v_1.3 in newborn neurons as well as mature neurons

**Fig 1:**
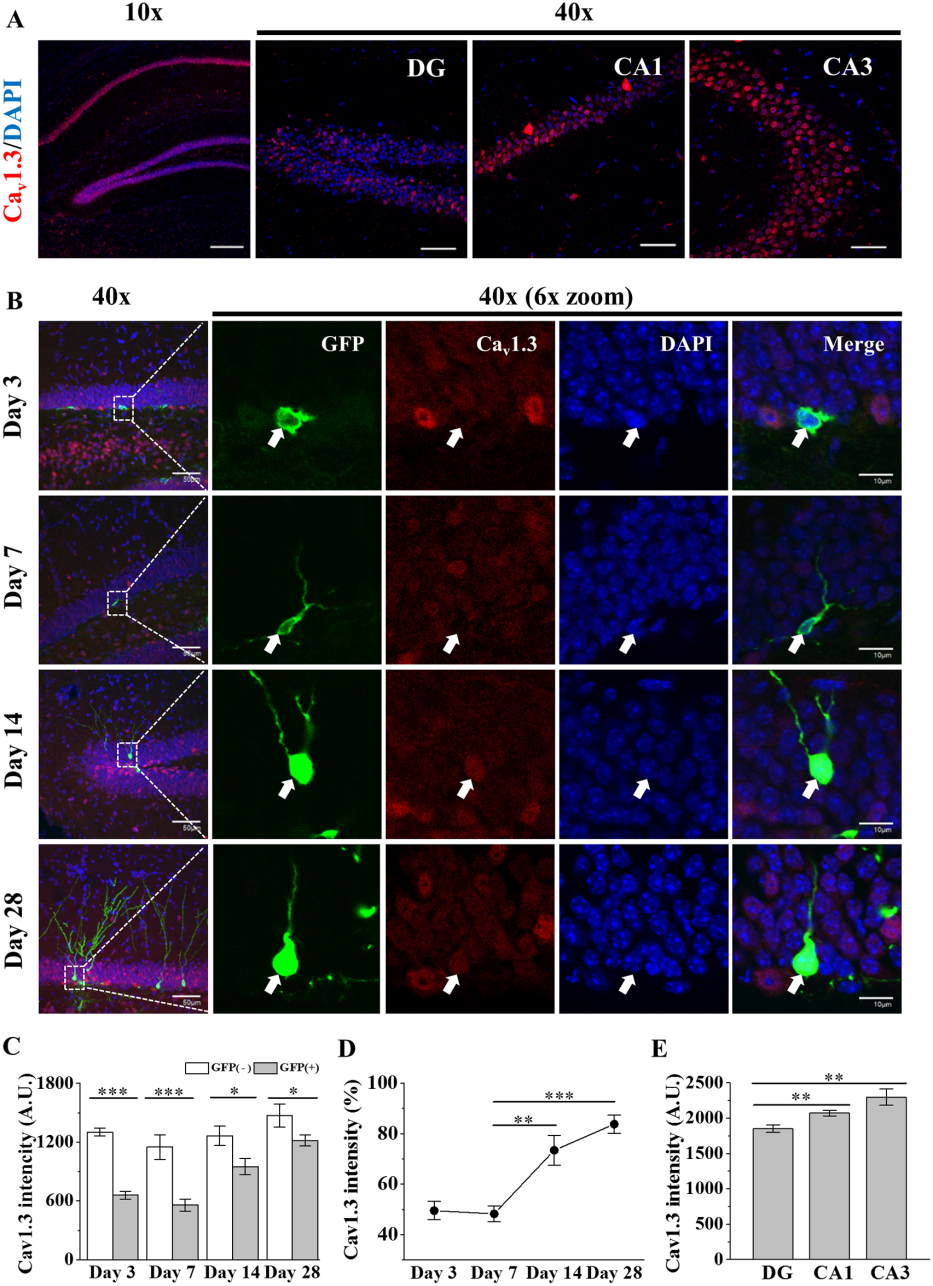
Expression of Ca_v_1.3 in adult hippocampal area. (A) Ca_v_1.3 expression in dorsal hippocampal area. Ca_v_1.3 is shown in red and DAPI, a nuclear maker, is shown in blue. *Scale bars*, 200 μm (10x) and 50 μm (40x). (B) Images of developmental profiling of Ca_v_1.3 expression in adult hippocampal newborn neurons. Confocal images of adult hippocampal newborn neurons, infected with GFP-retrovirus and stained with Ca_v_1.3 antibody (red), were taken at 3, 7, 14 and 28 days after infection. White arrows indicate newborn cells infected with retrovirus. *Scale bars*, 50μm (40x) and 10 μm (40x/6x-zoom). (C) Ca_v_1.3 antibody fluorescent intensity of newborn neurons (GFP (+), filled bar) and control mature neurons (GFP (-), open bar) of dorsal hippocampus shown at (B). A.U. indicates arbitrary unit. (Day 3, GFP(+), 658.10 ± 41.58, n = 9, GFP(-), 1302.51 ± 40.98, n = 50; Day 7, GFP(+), 558.19 ± 61.26, n = 9, GFP(-), 1149.03 ± 126.35, n = 50; Day 14, GFP(+), 950.79 ± 83.09, n = 7, GFP(-), 1264.75 ± 97.98, n = 50; Day 28, GFP(+), 1217.75 ± 55.34, n = 13, GFP(-), 1470.64 ± 115.84, n = 50; *p*(Day 3) < 0.000, *p*(Day 7) = 0.000, *p*(Day 14) = 0.035, *p*(Day 28) = 0.041). Two-way ANOVA, F_G_ = 66.17, *p* = 0.000; F_T_ = 15.22, *p* = 0.000; F_G+T_ = 3.20, *p* = 0.031. (D) Normalized Ca_v_1.3 antibody fluorescent intensity of newborn neurons to that of mature neurons. (Day 3, 49.52 ± 3.61%, n = 9; Day 7, 48.26 ± 3.08%, n = 9; Day 14, 73.42 ± 5.94%, n = 7; Day 28, 83.76 ± 3.58%, n = 13; *p*(Day 3–7) = 0.795, *p*(Day 7–14) = 0.001, *p*(Day 14–28) = 0.138). One-way ANOVA, F = 20.913, *p* = 0.000. (E) Comparison of Ca_v_1.3 expression among DG, CA1 and CA3 regions of dorsal hippocampus shown at (A) (each, n = 10). (DG, 1851.50 ± 54.44, n = 10; CA1, 2072.08 ± 38.63, n = 10; CA3, 2298.10 ± 115.40, n = 10; *p*(DG-CA1) = 0.004, *p*(CA1-CA3) = 0.080, *p*(DG-CA3) = 0.003). One-way ANOVA, F = 8.42, *p* = 0.001. *, **, *** indicate *p* < 0.05, *p* < 0.01, *p* < 0.001, respectively.

Ca_v_1.3 is expressed in various regions of the brain such as cortex, hippocampus, lateral ventricle, cerebellum, olfactory bulb, and thalamus [39, 40]. *In situ* results confirmed the broad existence of Ca_v_1.3 mRNA in mouse brains of embryo to adult (S1A Fig). Immunohistochemistry of Ca_v_1.3 showed the strong expression in cell body regions compared to dendritic and axonal regions of neurons in *Cornu Ammonis 1* (CA1), CA3 and DG of dorsal hippocampus and cortex of adult mice (Fig 1A and S1B Fig), which is consistent with Veng and Browning (2002) [41]. Previous study with a mouse line expressing Ca_v_1.3 tagged with eGFP showed co-labeling of Ca_v_1.3 with NeuN (+) cells and some of nestin (+) or GFAP (+) cells but little co-labeling with DCX (+) cells, suggesting a differential expression of Ca_v_1.3 during development of neural stem cells (NSCs) in DG [20]. To check how Ca_v_1.3 is expressed in newborn neurons of DG, GFP-retrovirus was injected to infect newborn cells. The results showed that Ca_v_1.3 expression of newborn neurons 3 to 7 days old was about half level of mature neurons and started to increase after Day 7 (Fig 1B and 1C). Ca_v_1.3 immuno-fluorescent intensities at 14 and 28 day old cells were increased by ~52% and ~74% over that of 7 day old cells, respectively (Fig 1D), suggesting that new Ca_v_1.3 expression was strongly triggered between 7 and 14 day old period. However, the fluorescent intensity of Ca_v_1.3 from cell bodies of newborn neurons of ≤ 28 days old was still significantly weaker than that of mature granule neurons (GFP (-) cells) (Fig 1D). Co-immunostaining of DCX and Ca_v_1.3 in WT mice shows that DCX (+) mature cells with tertiary dendrites have higher Ca_v_1.3 expression in the cell body than DCX (+) immature cells (S2A–S2C Fig). Within hippocampal regions, cell body Ca_v_1.3 intensity was stronger in CA3 region than those in CA1 and DG areas by ~10% and ~19%, respectively (Fig 1E). The results show that Ca_v_1.3 is expressed in both newborn cells and mature neurons and the expression is low initially and then after 7 days old keeps increasing until adult stage.

### Reduction of the survival rate of hippocampal newborn neurons in Ca_v_1.3 KO mice

**Fig 2:**
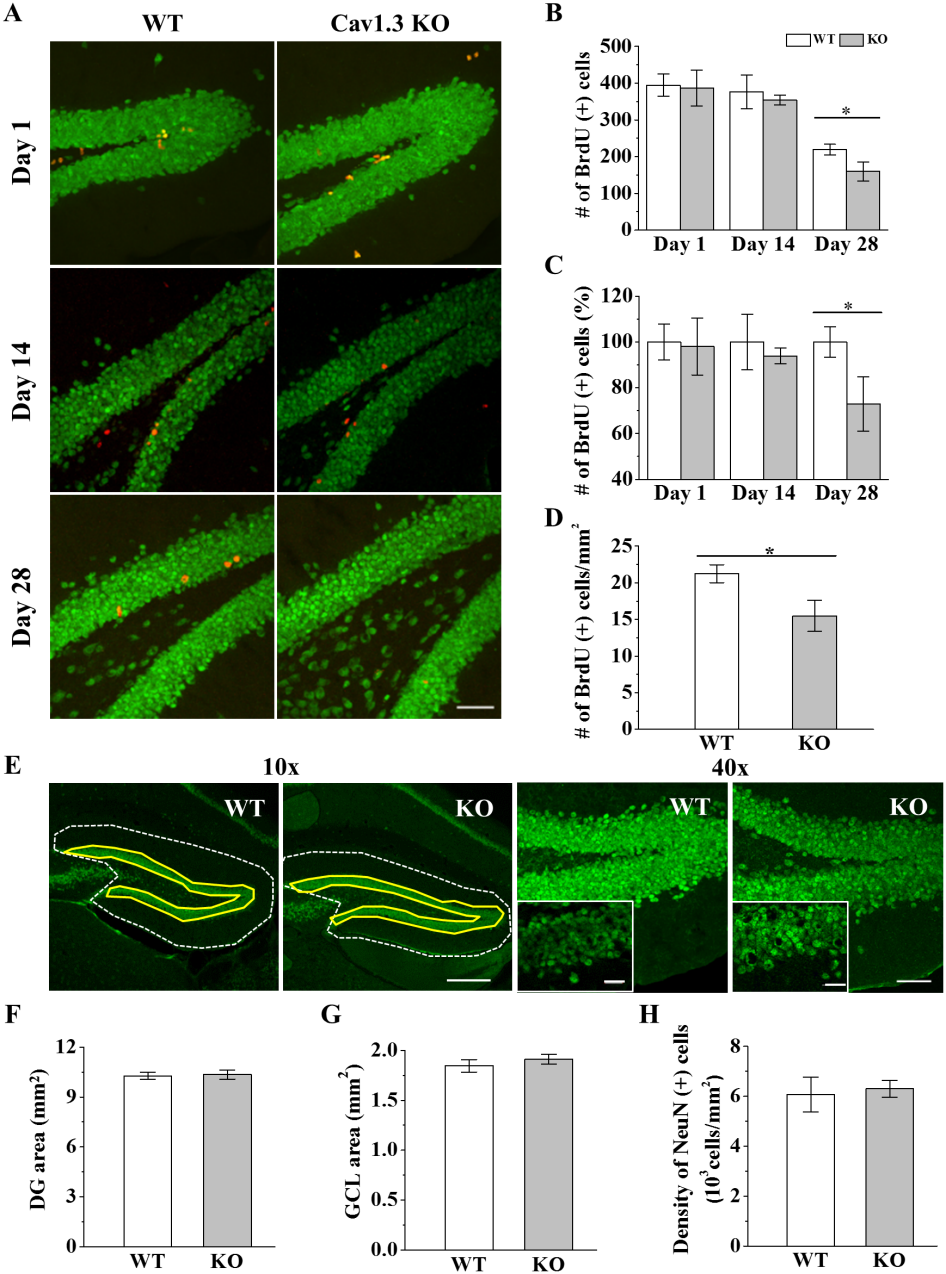
Proliferation and survival of DG newborn cells of dorsal hippocampus in Ca_v_1.3 KO mouse. (A) Confocal images of BrdU (+) cells (red) and NeuN (+) cells (green) in Ca_v_1.3 KO and WT mouse. Images are acquired at 1, 14 and 28 days after BrdU injection. *Scale bar*, 50 μm. (B) Number of BrdU (+) cells. (Day 1, WT, 394.667 ± 30.78 cells, n = 8; KO, 387 ± 49.05, n = 6, *p* = 0.660; Day 14, WT, 376.6 ± 45.85 cells, n = 6; KO, 35.8 ± 13.22 cells, n = 6, *p* = 0.472; Day 28, WT, 219 ± 13.61 cells, n = 7; KO, 159.83 ± 23.70 cells, n = 6, *p* = 0.046). * indicates *p* < 0.05. Two-way ANOVA, F_G_ = 3.80, *p* = 0.061; F_T_ = 59.12, *p* = 0.000; F_G+T_ = 0.84, *p* = 0.444. (C) Number of BrdU (+) cells of KO mice normalized to that of WT mice at given day. (Day 1, WT, 100 ± 7.80%, n = 8, KO, 98.06 ± 12.43%, n = 10; Day 14, WT, 100 ± 12.17%, n = 6, KO, 93.95 ± 3.50%, n = 6; Day 28, WT, 100 ± 6.73%, n = 7, KO, 72.98 ± 11.85%, n = 6, *p* = 0.046). Two-way ANOVA, F_G_ = 4.61, *p* = 0.040; F_T_ = 1.82, *p* = 0.179; F_G+T_ = 1.90, *p* = 0.168. (D) Number of BrdU (+) cells per DG area at Day 28. (WT, 21.24 ± 1.22 cells/mm^2^, n = 7; KO, 15.47 ± 2.12, n = 6, *p* = 0.032). (E) *Left*, example images for area measurements of DG (white dot line) and GCL (yellow line). *Right*, NeuN (+) cells (green) of DG in Ca_v_1.3 KO and WT mice. *Scale bars*, 100 um (10x), 50 μm (40x), 10 μm (*insets*, 40x/5x-zoom). (F) DG area (WT, 9.92 ± 0.19 mm^2^, n = 6, KO, 9.58 ± 0.18 mm^2^, n = 6, *p* = 0.833), (G) GCL area (WT, 1.85 ± 0.063 mm^2^, n = 6, KO, 1.91 ± 0.05 mm^2^, n = 7, *p* = 0.445) and (H) Density of NeuN (+) cells in GCL (WT, 6071 ± 691.88 cells/mm^2^, n = 11, KO, 6304.71 ± 339.34 cells/mm^2^, n = 12, *p* = 0.897).

A recent study reported that survival of adult newborn neurons 28 days old was reduced in Ca_v_1.3 KO mice [20]. However, it is unclear when the survival rate of newborn neurons in KO mice starts to change differentially. We first confirmed KO of Ca_v_1.3 (S1C–S1D Fig) and deafness of KO mouse (S1E Fig). To look into the time course of development of newborn cells, the number of DG newborn cells of dorsal hippocampus of KO mice was analyzed at following days after BrdU injection; 1 day for proliferation, 14 day for the early survival and 28 day for the late survival (Fig 2A). The newborn cell numbers at day 1 and day 14 were not different between WT and KO mice. At day 28, even in WT mice, the number of BrdU (+) cells was reduced by ~42% compared to that of day 14 (Fig 2B). In KO mice, the number of BrdU (+) cells at day 28 was further reduced in KO mice by ~27% compared to that of WT (Fig 2C), suggesting a contribution of Ca_v_1.3 on the survival of newborn neurons. The density of BrdU (+) cells per DG area at day 28 was also reduced in KO mice by ~27% (Fig 2D). Analysis of DCX (+) cells showed that the total number of DCX (+) cells was not changed in KO mice but percentage of mature cells selectively decreased in KO mice (S2D–S2F Fig). Works on the correlation between the survival of newborn cells and the area/volume of DG have been controversial [20, 34, 42]. Studies by Marshallinger et al. (2015) and Noto et al. (2016) showed that the change of the survival rate of newborn cells was positively related with that of the volume of DG in Ca_v_1.3 KO and 5-HT_1A_R overexpressing transgenic mice, respectively, but Lee et al. (2016) showed that the survival of newborn cell was increased in the absence of the change in DG area in Ca_v_1.2 forebrain KO mice. We measured DG area and the area and density of granule cells in KO mouse using NeuN antibody (see Methods). The results showed that areas of DG and granule cell layer and the density of mature granule cells were not significantly changed at day 28 in KO mice (Fig 2F–2H).

### Impairments of dendritic and MFB growth and MFB filopodia development of hippocampal newborn neurons in Ca_v_1.3 KO mice

**Fig 3:**
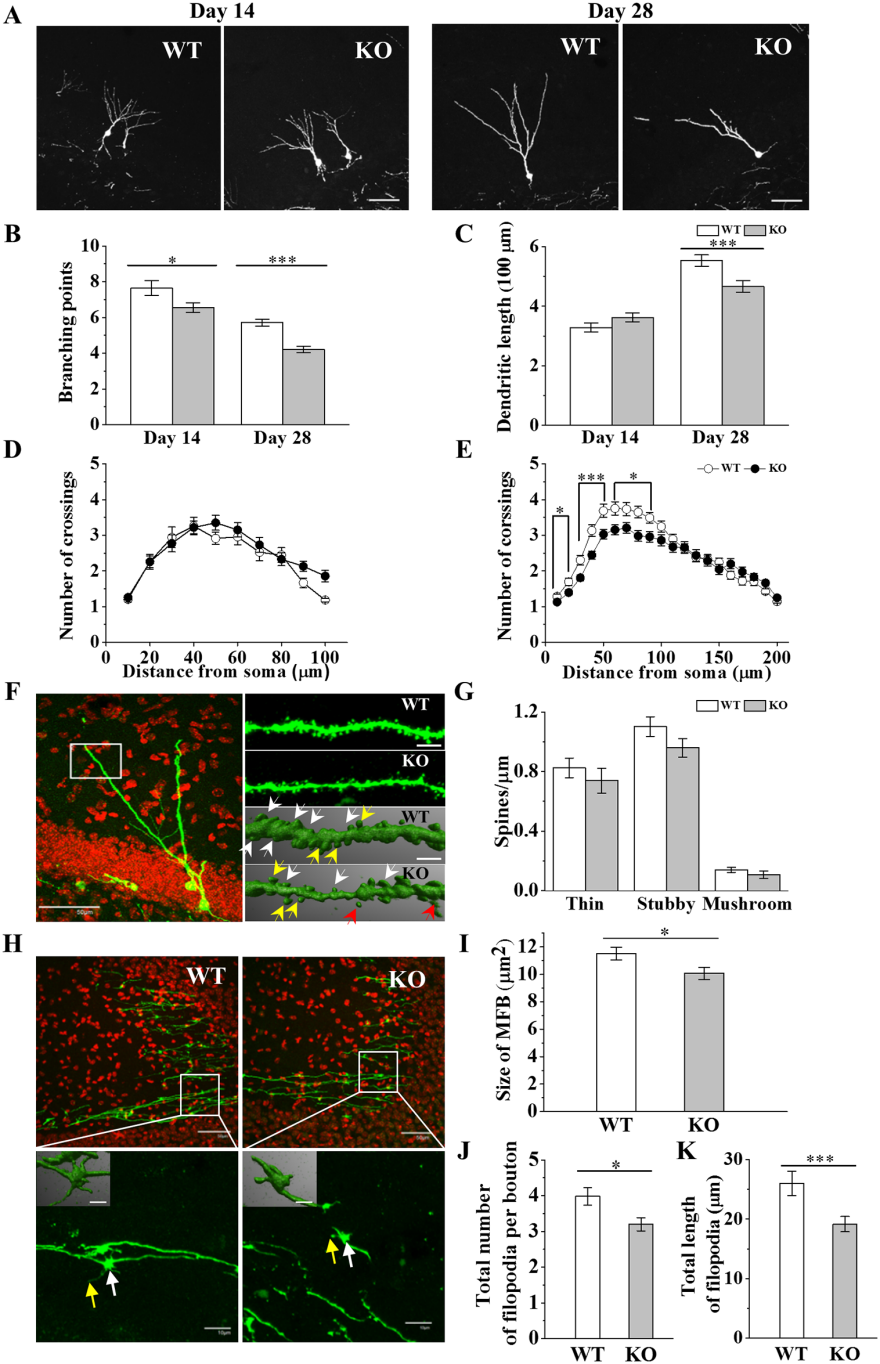
Effects of Ca_v_1.3 KO on developments of dendrites, spines and MFB filopodia of DG newborn neurons. (A) Confocal images of GFP (+) neurons at 14 and 28 days after GFP-retroviral infection. *Scale bar*, 50 μm. (B-E) Quantification of dendritic development. *, **, *** indicate *p* < 0.05, *p* < 0.01, *p* < 0.001, respectively. (B) Total number of dendritic branching points at 14 and 28 days after viral infection. (Day 14, WT, 7.64 ± 0.41, n = 62, KO, 6.55 ± 0.25, n = 102, *p* = 0.017; Day 28, WT, 5.71 ± 0.20, n = 107, KO, 4.20 ± 0.18, n = 120, *p* < 0.00001, n = 3 animals per group). Two-way ANOVA, F_G_ = 26.96, *p* = 0.000; F_T_ = 73.08, *p* = 0.000; F_G+T_ = 0.68, *p* = 0.001. (C) Total dendritic length measurement at 14 and 28 days after viral injection. (Day 14, WT, 328.35 ± 14.57 μm, n = 69, KO, 362.34 ± 45.06 μm, n = 100, *p* = 0.12; Day 28, WT, 552.90 ± 19.15 μm, n = 107, KO, 466.34 ± 19.97 μm, n = 119, n = 4 animals per group, *p* = 0.002). Two-way ANOVA, F_G_ = 1.96, *p* = 0.162; F_T_ = 76.56, *p* = 0.000; F_G+T_ = 10.31, *p* = 0.001. (D-E) Number of dendritic crossings in Sholl analysis at 14 (D) and 28 days (E) after viral infection. (Day 28: 10 μm, WT, 1.29 ± 0.07, KO, 1.13 ± 0.04, *p* = 0.022; 20 μm, WT, 1.70 ± 0.10, KO, 1.39 ± 0.07, *p* = 0.011; 30 μm, WT, 2.30 ± 0.13, KO, 1.81 ± 0.09, *p* = 0.001; 40 μm, WT, 3.13 ± 0.16, KO, 2.44 ± 0.11, *p* = 0.001; 50 μm, WT, 3.69 ± 0.18, KO, 3.03 ± 0.13, *p* = 0.004; 60 μm, WT, 3.75 ± 0.18, KO, 3.15 ± 0.14, *p* = 0.010; 70 μm, WT, 3.73 ± 0.19, KO, 3.21 ± 0.15, *p* = 0.025; 80 μm, WT, 3.65 ± 0.16, KO, 2.98 ± 0.14, *p* = 0.005; 90 μm, WT, 3.49 ± 0.15, KO, 2.96 ± 0.15, *p* = 0.013; WT, n = 107 cells, KO, n = 122 cells, n = 4 animals per group). Two-way ANOVA, F_G_ = 10.54, *p* = 0.001; F_T_ = 27.18, *p* = 0.000; F_D_ = 92.87, *p* = 0.000; F_G+T_ = 34.97, *p* = 0.000; F_G+D_ = 1.23, *p* = 0.27; F_T+D_ = 23.76, *p* = 0.000; F_G+T+D_ = 0.92, *p* = 0.504. (F) *Left*, representative image (60x) of newborn neurons at 28 days after GFP-retroviral infection. Red, DAPI. White rectangle shows a distal dendritic region of a newborn neuron of Ca_v_1.3 WT mice for spine analysis. *Right*, exemplary high magnification (60x/6x-zoom) images (*top*) and 3D reconstruction images (*bottom*) of a distal dendritic region of a newborn neuron of WT and Ca_v_1.3 KO mice. White arrows indicate stubby spines, yellow arrows indicate mushroom spines and red arrows indicate thin spines. *Scale bar*, 50 μm (60x), 5 μm (60x/6x-zoom) and 2 μm (3D image). (G) Spine density plot for each type of spines. (Thin spines, WT, 0.82 ± 0.07 spines/μm, KO, 0.83 ± 0.06 spines/μm, *p* = 0.434; stubby spines, WT, 1.10 ± 0.07 spines/μm, KO, 0.95 ± 0.05 spines/μm, *p* = 0.064; mushroom spines, WT, 0.14 ± 0.017 spines/μm, KO, 0.20 ± 0.06 spines/μm, *p* = 0.409, WT, n = 28 cells, KO, n = 29 cells, n = 2 animals per group). (H) *Top*, confocal images of CA3 region axonal fibers of newborn neurons at 28 days after GFP expressing retrovirus injection. Red, DAPI. *Bottom*, high magnification images of axonal boutons near CA3 pyramidal cell layer. White and yellow arrows indicate boutons and filopodia, respectively. *Insets*, 3D image of bouton and filopodia. *Scale bars*, 50 μm (40x), 10 μm (40x/6x-zoom), 5 μm (*insets*). (I) Size of mossy fiber boutons (WT, 11.52 ± 0.47, n = 84 boutons; KO, 10.07 ± 0.45, n = 70 boutons, n = 3 animals per group, *p* = 0.029). (J) Total number of filopodia of axonal boutons (WT, 3.98 ± 0.25, n = 53 boutons, KO, 3.2 ± 0.19, n = 65 boutons, n = 3 animals per group, *p* = 0.010) and (K) the length of filopodia of axonal boutons (WT, 25.99 ± 2.02 μm, n = 53 boutons, KO, 19.16 ± 1.29 μm, n = 65 boutons, n = 3 animals per group, *p* = 0.004).

Adult hippocampal newborn neurons grow substantially during 14 to 28 day old period after mitosis [36]. The correlation of this period with the time for survival or synaptic integration of newborn neurons in DG has been suggested [32, 43, 44]. In this study, we have adopted retrovirus labeling to analyze morphological development of dendrites and MFB, MFB filapodia and dendritic spines of newborn neurons of dorsal hippocampus of Ca_v_1.3 KO mice [36]. Exemplary images of GFP (+) dendritic processes of newborn neurons were shown in Fig 3A. The numbers of total branching points of dendrites of newborn neurons at day 14 and day 28 in KO mice were significantly reduced by ~14% and ~26%, respectively, compared with those of WT mice (Fig 3B). The total dendritic length of newborn neurons at day 28 was also significantly reduced by ~16% in KO mice (Fig 3C). To examine the dendritic complexity of newborn neurons, Sholl analysis was applied with Fiji program [45] (Fig 3D and 3E). The dendritic complexity within range of 100 μm from soma of newborn neurons at day 28 was significantly reduced by 12 ~ 22% in KO mice (Fig 3E). These results together suggest that both the appearance of initial dendrites from the soma and the activity of branching and growth of dendrites of newborn neurons in KO mouse seem quite normal until 14 days old, when most dendrite length is shorter than 100 μm, but when it is over 14 days old, the appearance of new dendrites from the soma or the activity of their branching and growth gets slower or inhibited although preexisting dendrites seem to keep growing normally over 100 μm long in KO mice. It is possible that higher expression of Ca_v_1.3 in newborn neurons ≥ 14 days old could be related with the later dendritic development.

During functional synapse formation, the morphology of spines might change from thin filpodia to stubby to mature mushroom types [36, 46]. LTCCs have been reported to contribute to calcium signaling in dendritic spines [47, 48]. However, it is largely unknown about roles of LTCCs in dendritic spine formation. Recently, it has been shown that splice variants of Ca_v_1.3 regulated the morphology of dendritic spines of cultured hippocampal neurons [49]. Therefore, to check an effect of Ca_v_1.3 in the spine development of adult newborn neurons *in vivo*, morphological types of dendritic spines were analyzed (Fig 3F). The results showed that the density of thin, stubby, mushroom types of spines of adult newborn neurons at day 28 was not changed significantly (Fig 3G). The result suggests that the effect of Ca_v_1.3 deletion of newborn neurons on spine development marginally occurs over 14 days old, if any, when functional dendritic spine formation occurs usually, further coinciding with the survival period of newborn neurons [36].

Newborn neurons also generate axonal fibers and make synaptic contacts with target neurons including hilar interneurons, CA3 pyramidal cells and interneurons when they are 17 to 28 days old [50–53]. Synapse formation could contribute to the dendritic maturation of newborn neurons [44, 54]. While the filopodia of the mossy terminals interact mainly with the GABAergic interneurons, MFBs form synapses on excitatory pyramidal cells and hilar mossy cells [55]. To find a role of Ca_v_1.3 on axonal development and possibly synapse formation, filopodia and the size of axonal boutons of newborn neurons in CA3 regions at day 28 were characterized in KO mice (Fig 3H). The size of MFB was decreased by ~13% in KO mice (Fig 3I). The total number and total length of filopodia per bouton were significantly smaller in KO mice by ~25% and ~27%, respectively (Fig 3J and 3K).

The result of morphological analysis of newborn neurons suggests that Ca_v_1.3 is mainly necessary for the proper development of both dendrite and axonal fibers and might contribute to the formation of functional synapses and thereby, possibly for the survival of newborn neurons.

### Shock sensitivity, locomotion, anxiety level, visual function and working memory seem normal in Ca_v_1.3 KO mice

**Fig 4:**
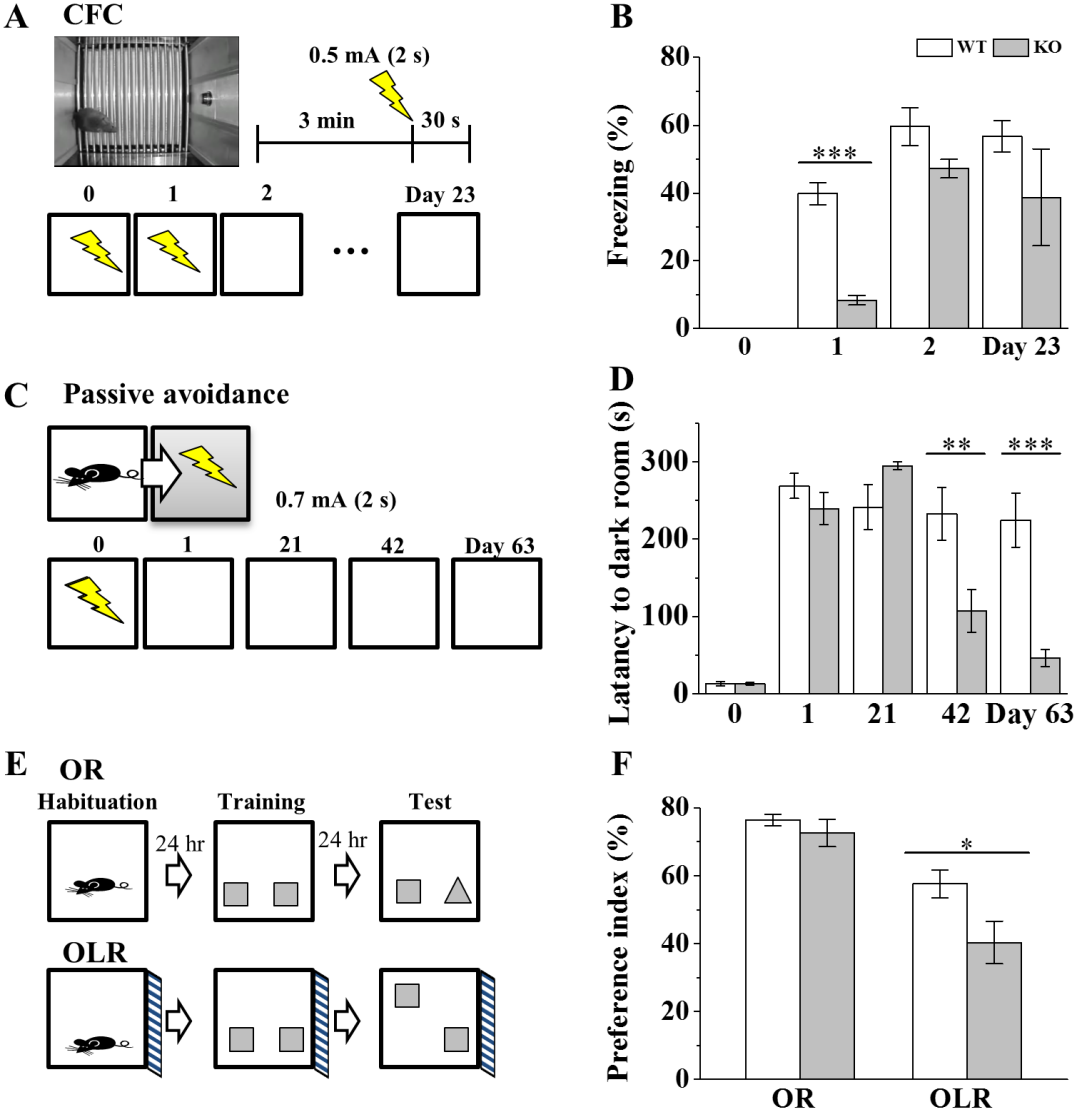
Impairments of hippocampus-dependent memory tasks in Ca_v_1.3 KO mice. (A) Scheme of CFC learning and memory tests. Both recent and remote CFC memories were assessed in the same chamber at Days 0, 1, 2 and 23. (B) Freezing responses of CFC memory tasks. (Day 0, WT, 0 ± 0, n = 5, KO, 0 ± 0, n = 5; Day 1, WT, 39.80 ± 3.33%, n = 15, KO, 8.39 ± 1.40%, n = 11, *p* < 0.00001; Day 2, WT, 59.59 ± 5.60%, n = 13, KO, 47.18 ± 2.74%, n = 9, *p* = 0.098; Day 23, WT, 56.75 ± 4.58%, n = 10, KO, 38.74 ± 14.30%, n = 6, p = 0.305). *, **, *** indicate *p* < 0.05, *p* < 0.01, *p* < 0.001, respectively, unless otherwise mentioned. Two-way ANOVA, F_G_ = 18.24, *p* = 0.000; F_T_ = 17.88, *p* = 0.000; F_G+T_ = 2.31, *p* = 0.106. (C) Scheme of PA tasks. (D) Latency of entrance to dark room of PA tasks. (Day 0, WT, 13.13 ± 2.88 s, n = 24, KO, 13.12 ± 1.75 s, n = 24, *p* = 0.990; Day 1, WT, 268.89 ± 16.18 s, n = 24, KO, 239.34 ± 20.72 s, n = 24, *p* = 0.267; Day 21, WT, 241.38 ± 28.90 s, n = 13, KO, 294.95 ± 5.05 s, n = 13, *p* = 0.080; Day 42, WT, 232.67 ± 34.10 s, n = 9, KO, 107.22 ± 27.86 s, n = 12, *p* = 0.010; Day 63, WT, 224.59 ± 34.76 s, n = 7, KO, 46.29 ± 10.71 s, n = 10, *p* = 0.000). Two-way ANOVA, F_G_ = 2.45, *p* = 0.12; F_T_ = 17.47, *p* = 0.00; F_G+T_ = 2.33, *p* = 0.08. (E) Schemes of OR and OLR tasks. (F) Preference index measurement of OR/OLR tasks. (OR task: WT, 76.41 ± 1.66%, n = 11, KO, 72.54 ± 4.0%, n = 9, *p* = 0.339; OLR task: WT, 55.19 ± 4.04%, n = 11, KO, 42.89 ± 4.13%, n = 9, *p* = 0.048).

Ca_v_1.3 is expressed in various cell types of mouse organs such as retina, inner hair cells, heart and pancreas [39, 56–59]. Therefore, to check whether null KO of Ca_v_1.3 in mouse might cause any serious neurobiological effects, some critical neurological screening tests were done before executing behavioral experiments. First of all, we confirmed the deafness of KO mice (S1E Fig) shown in [35, 60], which made us to use CFC rather tone-fear conditioning. Second, to examine the sensitivity to electrical shocks, responses to shocks were categorized such as flinch, vocalization and jump after the shock (S3A Fig) [61]. The results showed for the first time that thresholds of responses to shocks in all three categories were not significantly different between KO and WT mice, indicating the normal sensitivity to shocks of KO mice. Third, the effect on body weight in Ca_v_1.3 KO mice has been debated [16, 60]. We found body weights of KO mice were slightly less by ~7% in 8-week old KO mice (S3B Fig), and the biological significance of the difference was not pursued. Fourth, to measure both the locomotor activity and the anxiety level, open field test was made. The results showed no significant difference in the total moving distance and in the ratio of the moving distance within the center area over the total moving distance (S3C and S3D Fig), indicating normal locomotor activity and anxiety level in KO mice. Fifth, to test the working memory, Y-maze test was applied. The total number of entries and the percentage of spontaneous alternation were not significantly different between KO and WT mice, suggesting normal working memory capability of KO mouse (S3E and S3F Fig). Sixth, in case of vision, results of OR test suggested that Ca_v_1.3 KO mouse has normal visual function (Fig 4E and 4F). These results of neurological screening along with the result of OR task suggest that deletion of Ca_v_1.3 would not significantly affect electrical sensitivity, locomotor activity, anxiety level, working memory performance and visual function of KO mouse, consistent with previous studies [16, 60, 62], but see McKinney et al. (2008) [18].

### Impairment of hippocampal-dependent memory tasks in Ca_v_1.3 KO mice

To investigate a role of Ca_v_1.3 in hippocampal-dependent memory tasks, Ca_v_1.3 KO mice were used for CFC, PA and OLR/OR learning tests. In case of recent CFC memory, it was shown that consolidation of one-trial CFC was impaired in KO mice but double-trial CFC was not [16]. In this study, both recent (Day 1) and remote (≥ Day 23) memory of CFC was investigated in KO mice (Fig 4A). The results showed that the recent CFC memory was impaired significantly in KO mice while the remote memory was not (Fig 4B). The result with KO mice suggests that lack of Ca_v_1.3 in mice may cause the impairment of recent CFC memory, consistent with McKinney and Murphy (2006) [16]. PA learning test showed that the remote memories at Day 42 and Day 63 of PA were impaired in KO mice by ~54% and ~80%, respectively, compared to WT mice but memory at Day 1 or 21 were normal in both groups (Fig 4C and 4D), consistent with the results of Pan et al. (2012) [38]. In case of OLR/OR tasks, OLR memory is more hippocampal-dependent than OR memory [21, 63, 64] and the recent memory of OLR task was examined because OLR task is reliable only in the recent memory task [65]. The result showed that OLR task was impaired in the recent memory performance but OR task was not in KO mice (Fig 4E and 4F). The result suggests that OLR memory processes are more sensitive to the hippocampal Ca_v_1.3 function.

### Impairment of both recent and remote CFC memories in dorsal hippocampal AAV-Ca_v_1.3 KD mice

**Fig 5:**
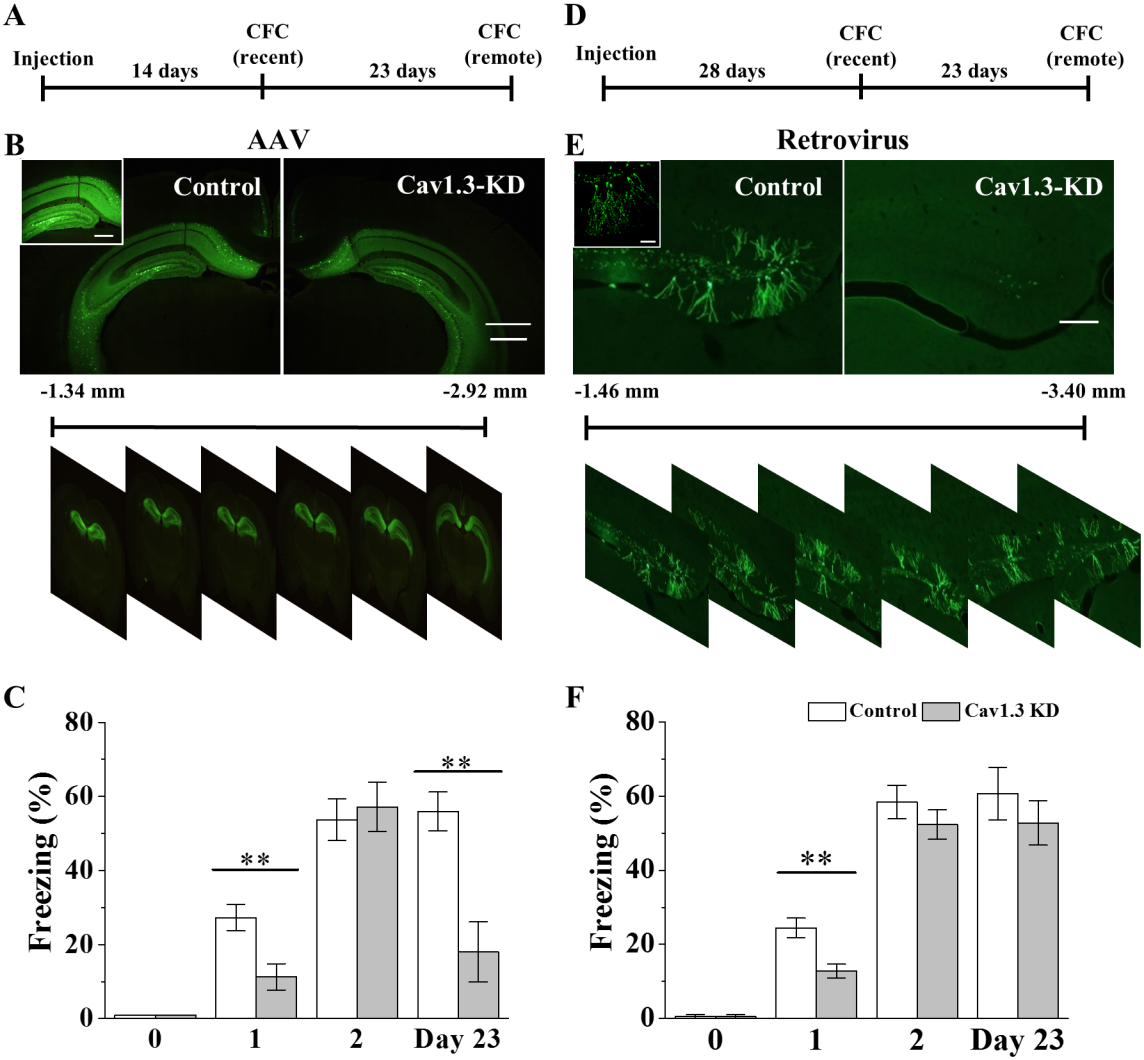
Effects of AAV- and retrovial-Ca_v_1.3 KD on both recent and remote memories of CFC. (A) Experimental scheme of recent and remote memory tests of CFC using AAV mediated Ca_v_1.3 KD in dorsal hippocampus. (B) *Top*, Representative images of GFP expression of AAV-Ca_v_1.3 KD cells in dorsal hippocampus. *Scale bars*, 500 μm and 200 μm (*insets*). *Bottom*, representative images of expression of GFP (+) AAV-Ca_v_1.3 KD control into the dorsal hippocampus of F1 mouse at 2 week of infection. (C) Freezing responses in AAV-Ca_v_1.3 KD and control mice. (Day 0, Control, 0 ± 0, n = 5, KD, 0 ± 0, n = 5; Day 1, Control, 27.25 ± 3.57%, n = 19, KD, 11.31 ± 3.55%, n = 17, *p* = 0.003; Day 2, Control, 53.74 ± 5.58%, n = 8, KD, 57.13 ± 6.65%, n = 7, *p* = 0.697; Day 23, Control, 55.96 ± 5.20%, n = 8, KD, 18.00 ± 8.11%, n = 7, *p* = 0.001). ** indicates *p* < 0.01. (D) Experimental scheme of recent and remote memory tests of CFC using retrovirus mediated Ca_v_1.3 KD in dorsal hippocampus. Two-way ANOVA, F_G_ = 15.09, *p* = 0.000; F_T_ = 27.49, *p* = 0.000; F_G+T_ = 6.12, *p* = 0.004. (E) *Top*, Representative images of GFP expression of retroviral-Ca_v_1.3 KD cells in DG of dorsal hippocampus at 28 days after infection. *Scale bars*, 200μm and 50μm (*insets*). *Bottom*, representative images of expression of GFP (+) retrovirus-Ca_v_1.3 KD control into the dorsal hippocampus of F1 mouse at 4 week of infection. (F) Freezing responses in retroviral-Ca_v_1.3 KD and control mice. (Day 0, Control, 0 ± 0, n = 5, KD, 0 ± 0, n = 5; Day 1, Control, 24.46 ± 2.63%, n = 14, KD, 12.80 ± 1.88%, n = 15, *p* = 0.003; Day 2, Control, 58.40 ± 4.54%, n = 14, KD, 52.33 ± 3.96%, n = 15, *p* = 0.321; Day 23, Control, 60.66 ± 7.09%, n = 9, KD, 52.78 ± 6.00%, n = 9, *p* = 0.409). Two-way ANOVA, F_G_ = 4.33, *p* = 0.041; F_T_ = 32.07, *p* = 0.000; F_G+T_ = 0.14, *p* = 0.866.

Results of KO mice study indicate that Ca_v_1.3 is necessary for the memory of hippocampal-dependent learning tasks but they cannot tell the type and location of cells involved. To identify regions of hippocampus where neuronal Ca_v_1.3 plays a role in CFC learning and memory, GFP-AAV containing Ca_v_1.3 KD shRNA and control GFP-AAV were made (S4A–S4C Fig) and injected into DG area of dorsal hippocampus (Fig 5B; S4D and S5A–S5C Figs). The results showed that KD of Ca_v_1.3 of infected neurons in dorsal hippocampus reduced both recent and remote memories of CFC by ~59% and ~68%, respectively (Fig 5C). Compared with KO mice data, significant suppression of remote CFC memory was observed in mice where KD occurred in dorsal hippocampal area only. Interestingly, CFC memory of intermediate period (Day 2) was normal in KD mice [16], further suggesting that the contribution of Ca_v_1.3 or the mechanism of CFC memory process might change dynamically along time [66]. The results suggest that Ca_v_1.3 in mature neurons of dorsal hippocampus is important for both recent and remote memories of CFC learning and Ca_v_1.3 activity from some group of neurons is enough for maintaining these CFC memories. Moreover, KD of Ca_v_1.3 in ventral hippocampal area by shRNA AAV did not impair the recent CFC memory (S4E and S4F Fig), suggesting that CFC learning and memory might use neuronal circuits of dorsal hippocampus mainly [67]. Another possibility is that the current KD method cannot affect all the ventral hippocampal neurons which are critically involved in CFC memory.

### Impairment of recent CFC memory in dorsal hippocampal retroviral-Ca_v_1.3 KD mice

The relationship between neurogenesis and memory tasks seems complex. In PA task, Pan et al. (2012) [38] showed that the remote PA memory was more dependent upon adult hippocampal neurogenesis than the recent memory. In case of OR/OLR tasks, OLR memory was dependent on adult hippocampal neurogenesis but OR memory was not [21, 63]. In spatial memory, both recent and remote memories were inhibited when adult hippocampal neurogenesis was impaired [21, 38, 68]. Since Ca_v_1.3 KO mouse has reduced survival rate of newborn neurons, it is plausible that the inhibitory effect on either recent memories of CFC and OLR or remote PA memory might be also due to the impaired neurogenesis in KO mice. To test this idea on CFC memory task, retrovirus-mediated KD method was adopted for gene regulation in proliferating neurons [36, 69]. To identify a role of Ca_v_1.3 in adult newborn neurons, GFP-Ca_v_1.3 shRNA-retrovirus and KD control GFP-retrovirus were generated and injected into DG area of dorsal hippocampus (Fig 5D; S6A and S6B Fig). The results showed that recent CFC memory was impaired in KD mice but remote CFC memory was not (Fig 5F). In KD mouse, bright GFP (+) neurons were rarely detected at ≥ 4 week of infection (≥ 10 animals). It is possible that infected newborns neurons might die out during the infection period, further convincing the positive effect of Ca_v_1.3 on survival of newborn neurons during that period (Fig 1). The results suggest that Ca_v_1.3 of newborn neurons in DG of dorsal hippocampus plays a critical role in the recent memory of CFC learning, possibly by helping the survival of newborn neurons, and some newborn neurons of dorsal hippocampal area might be enough for performing the CFC memory task.

## Discussion

It has been reported that Ca_v_1.3 may regulate survival of adult newborn neurons and hippocampal-dependent memory such as CFC [16, 20]. However, it is unclear how Ca_v_1.3 is involved in adult neurogenesis process and what regional neurons of hippocampus are sufficiently related with hippocampal-dependent learning & memory tasks. This study focused on roles of Ca_v_1.3 in the development of newborn neurons using KO mice and on the regional role of Ca_v_1.3 within hippocampus in learning & memory tasks using KD strategy. Functions of Ca_v_1.3 of mature or immature neurons in learning & memory were tried to be differentiated with AAV- vs retroviral KD methods. We show that Ca_v_1.3 plays a role for proper developments of dendrites, MFBs and filopodia of MFBs of adult newborn neurons during maturation and confirm the reduction of survival of newborn neurons in Ca_v_1.3 KO mice. We further show that various hippocampal-dependent memory tasks are impaired in KO mice and AAV KD of Ca_v_1.3 only in some of dorsal hippocampal neurons seems enough to impair CFC memory. Meanwhile, AAV KD in ventral hippocampal area has little effect on CFC memory. Moreover, retroviral KD mice study reveals a function of Ca_v_1.3 of dorsal hippocampal newborn cells in the recent CFC memory.

### Endogenous expression of Ca_v_1.3 during development of newborn neuron overlaps with the period of survival of newborn cells

Ca_v_1.3 is expressed in hippocampus [1, 10, 70]. LTCC blockers have reduced the survival and differentiation of late stage newborn neurons *in vitro* and *in vivo* [30, 31, 33, 71]. Recent Ca_v_1.3 KO mouse studies showed the reduction of survival of newborn neurons in adult hippocampus [20, 34]. Co-expressions of Ca_v_1.3 with neurogenesis stage makers such as nestin, PCNA, DCX, NeuN and GFAP have been shown in adult hippocampus [20]. However, it is still unclear how Ca_v_1.3 of newborn neurons is regulated and what functions it is related with. In this study, we find that Ca_v_1.3 expression in adult newborn cells starts to increase during maturation stage (≥ day 14) of development (Fig 1C and 1D), which coincides in general with the fate decision stage of newborn cells [72]. Moreover, we further show that the reduction of survival of newborn neurons occurs mostly around 28 days after mitosis in both WT and KO mice (Fig 2A–2D), which overlaps with the time when Ca_v_1.3 expression in newborn neurons becomes near the level of mature neurons (Fig 1D). In addition, the reduction of BrdU (+) cells at day 28 even in WT mouse suggests that Ca_v_1.3 is only a part of endogenous survival process of newborn neurons. It will be interesting to know whether overexpression or faster expression of Ca_v_1.3 in newborn neurons during early developmental period could inhibit or slow down the reduction of survival rate. Recently Kruger et al. (2017) developed a Ca_v_1.3 overexpressing transgenic mouse for normal aging model, which can be used for testing these possibilities [73]. In summary, the correlation of the period of enhanced Ca_v_1.3 expression in WT mice with the reduction of survival of adult newborn neurons in KO mice suggests an endogenous function of Ca_v_1.3 in the survival process of newborn neurons. For comparison, Ca_v_1.2 forebrain KO also showed the reduction of survival of newborn neurons [38]. The exact functional difference of Ca_v_1.2 and Ca_v_1.3 in the newborn neuron survival process remains to be studied.

### Ca_v_1.3 is necessary for the development of dendrites, MFBs and MFB filopodia of newborn neurons and for their survival

Newborn neurons derived from NSCs of DG need to develop proper dendrites and axonal terminals for synaptogenesis, while avoiding cellular death pathways, and thereby timely synaptic integration into preexisting neural networks, which is necessary for the functional maturation and survival [53]. LTCCs have been implicated in the neurite outgrowth of immature neuronal cell lines and cultured hippocampal or cortical neurons [74–80]. However it is unclear how LTCC isoforms are involved during neuronal development. Ca_v_1.2 is observed in pioneer axons of developing forebrain [81] and Ca_v_1.3 KO mice shows the reduction of axon arbor morphology in auditory brainstem at P10-12 [82]. We find that the development of MFB filopodia near CA3 as well as the growth of dendrites of DG newborn neurons is impaired in Ca_v_1.3 KO mice (Fig 3). Lesser growth of dendrites and axonal filopodia might result in the less formation of functional synapses of newborn neurons when it is time for synaptic integration to occur.

The positive relationship between neuronal outgrowth and survival of newborn neurons has been suggested. Impairments of both survival and neurite outgrowth of newborn neurons were observed [83–86] and enhancement of neurite outgrowth of newborn neurons was associated with their survival *in vivo* [87]. Furthermore, the correlation of reduction of survival and spine density in adult newborn neurons *in vivo* was also shown [84, 88, 89]. When spines get mature, it changes morphology from thin filopodia to stubby to mushroom shape, reflecting a maturation stage or functional difference [46]. The result shows that during the early stage of development, most spines of newborn neurons are either thin filopodia or stubby type and mushroom type is a few, which is consistent with the expectation when functional synapses have not yet been actively formed. Our result suggests that Ca_v_1.3 might be involved in the stability or formation of stubby spines and Ca_v_1.3 deletion would reduce the functional synapse formation and survival of newborn neurons, resulting in the impairment of hippocampal-dependent learning and memory tasks.

The relationship between the survival of adult newborn neurons and the volume/area of DG has been controversial [20, 34]. Marschallinger et al. (2015) has described the correlation of the reduction of DG volume and the survival of adult neurogenesis in Ca_v_1.3 KO mice [20]. Meanwhile, our results show that areas of DG GCL as well as DG were not changed though newborn cell survival is reduced in Ca_v_1.3 KO mice. This inconsistence may be caused by difference of age of animals or quantification methods. Recently, Lee et al. (2016) [34] have reported no change of DG GCL thickness in forebrain Ca_v_1.2 cKO mice that exhibit the reduction of survival of adult newborn neurons. The clarification of the relationship between the total DG cell number and the neurogenesis activity remains to be studied along the age of mouse.

### Ca_v_1.3 in mature or immature neurons of dorsal DG regions plays differential roles in CFC memory processes

Role of LTCCs in the consolidation of CFC has been reported using pharmacological and genetic methods [11, 13, 16, 90] and DG-CA3 regions of dorsal hippocampus are suggested for their involvement in contextual memory using lesion or gene deletion methods [66, 91]. McKinney et al. (2006, 2009) has described significant impairment in the consolidation of recent CFC memory without impairment in the extinction of Ca_v_1.3 KO mice [3, 16]. Studies of conventional KO mice did not tell what brain region, such as dorsal vs ventral hippocampus, is important for the memory of CFC. Some electrolytic and pharmacological lesion studies suggest dorsal hippocampus as a functional region for CFC [92–96] and others relate ventral hippocampus with CFC memory [97–99]. In this study, we showed virus-mediated localized effect of Ca_v_1.3 KD on CFC memory for the first time. The impairment of both recent and remote memories of CFC learning was observed in mice where AAV-Ca_v_1.3 KD occurs in DG-CA3 regions of dorsal hippocampus (Fig 5A; S4D Fig). On the contrary, the recent CFC memory was normal when AAV KD occurs in ventral hippocampal area (S4E and S4F Fig), suggesting a dominant role of Ca_v_1.3 in the dorsal hippocampal circuitry in the recent CFC memory process.

Regarding role of newborn neurons in learning and memory process, it was shown that enhancement of hippocampal neurogenesis improved the learning and memory tasks such as CFC, OR, PA and water-maze [23, 100, 101]. And ablation of hippocampal neurogenesis using x-ray irradiation or pharmacological or transgenic methods impaired CFC learning [24–26, 102]. It has been suggested that Ca_v_1.3 and adult neurogenesis are related with OLR memory [20] and 4 to 6 week old newborn neurons are involved in CFC and OR tasks using x-ray irradiation [28]. Here, by applying retrovirus-mediated Ca_v_1.3 KD in dorsal hippocampal area for the first time, we revealed the impairment of recent CFC memory when KD was maintained for 4 weeks (Fig 5F).

Our results suggest that Ca_v_1.3 channel of adult dorsal hippocampal neurons, either immature or mature, has endogenous functions in CFC memory process. Relating to Ca_v_1.2, deletion in forebrain Ca_v_1.2 had no effect on the consolidation and extinction of CFC [18] but was critical on the remote memory of water-maze [17]. Therefore, it seems that both Ca_v_1.2 and Ca_v_1.3 do have differential roles depending on the kind of learning and memory task.

### Role of Ca_v_1.3 in PA, OR and OLR task

LTCCs antagonists have produced controversial results about the role of LTCCs in PA task. LTCC blockers such as verapamil or nimodipine induced either impairment or enhancement of PA [103–106]. We find that KD of Ca_v_1.3 in dorsal hippocampal neurons inhibits the remote PA memory. In case of OR and OLR tasks, our results suggest that Ca_v_1.3 has a role in the recent OLR memory but not in OR task, consistent with Marschallinger et al. (2015) [20]. However, Pan et al. (2012) has described that the inhibition of adult neurogenesis induced by deletion of ERK5 is associated with the impairment in the memory at 48 hours after training of OR task [38]. These results indicate that there might be diverse mechanisms for OR or OLR memory process in terms of involvement of neurogenesis and type of LTCC isoforms or depending on the memory duration.

In this study, we have shown that Ca_v_1.3 in dorsal hippocampal neurons is involved in the development and survival of newborn neurons and in hippocampal-dependent learning and memory tasks. However, the mechanism of how Ca_v_1.3 contributes to the survival or death of newborn cells during development remains to be studied. Our results do not tell whether the effect of Ca_v_1.3 removal on the survival and development of newborn cells is cell-autonomous effect or not. Therefore, it will be interesting to know whether Ca_v_1.3 mediated Ca^2+^-dependent survival of newborn neurons is cell-autonomous process or not. Activity-dependent modulation of adult hippocampal neurogenesis suggests that extrinsic factors such as GABA or neuropeptides as well as synaptic activity might also be involved in the survival of newborn neurons [30, 107, 108]. Newborn neurons are derived from NSCs which are originated from glial cells [109, 110]. As a supplier of extrinsic niche environments, glial cells are known to release neurotransmitters such as glutamate and GABA as well as diverse cytokines and nitric oxide through intracellular Ca^2+^-dependent or—independent ways [111]. Since Ca_v_1.3 is expressed in nestin (+) NSCs and Ca_v_1.3 deletion leads to a decrease of hippocampal neurogenesis and of GFAP (+) area in 3-month old mice, it will be important to further clarify the role of Ca_v_1.3 during transitions from glia-like NSCs to newborn neurons to mature neurons.

## Supporting information

S1 Fig Characterization of Ca_v_1.3 KO mice. (TIF)

S2 Fig Expression of Ca_v_1.3 and DCX of dorsal hippocampus in Ca_v_1.3 WT and KO mice. (TIF)

S3 Fig Neurological screening tests in Ca_v_1.3 KO mice. (TIF)

S4 Fig Characterization of AAV-mediated Ca_v_1.3 KD *in vitro* and *in vivo*. (TIF)

S5 Fig Comparison of GFP expressions at dorsal and ventral hippocampus mediated by AAV-Ca_v_1.3 KD injections, respectively. (TIF)

S6 Fig Characterization of retrovirus-mediated GFP (+) Ca_v_1.3 KD *in vitro* and *in vivo*. (TIF)

S1 File A role of Cav1.3 channels in behavior for POne-SI-v11. (DOCX)

S2 File NC3Rs ARRIVE guidelines checklist (fillable)-chkim-v1. (PDF)
