# Supplementary figures and images for: Reduction of Cav1.3 channels in dorsal hippocampus impairs the development of dentate gyrus newborn neurons and hippocampal-dependent memory tasks
Source: PLoS One. 2017 Jul 17;12(7):e0181138. doi: 10.1371/journal.pone.0181138 (PMC5513490; doi:10.1371/journal.pone.0181138)

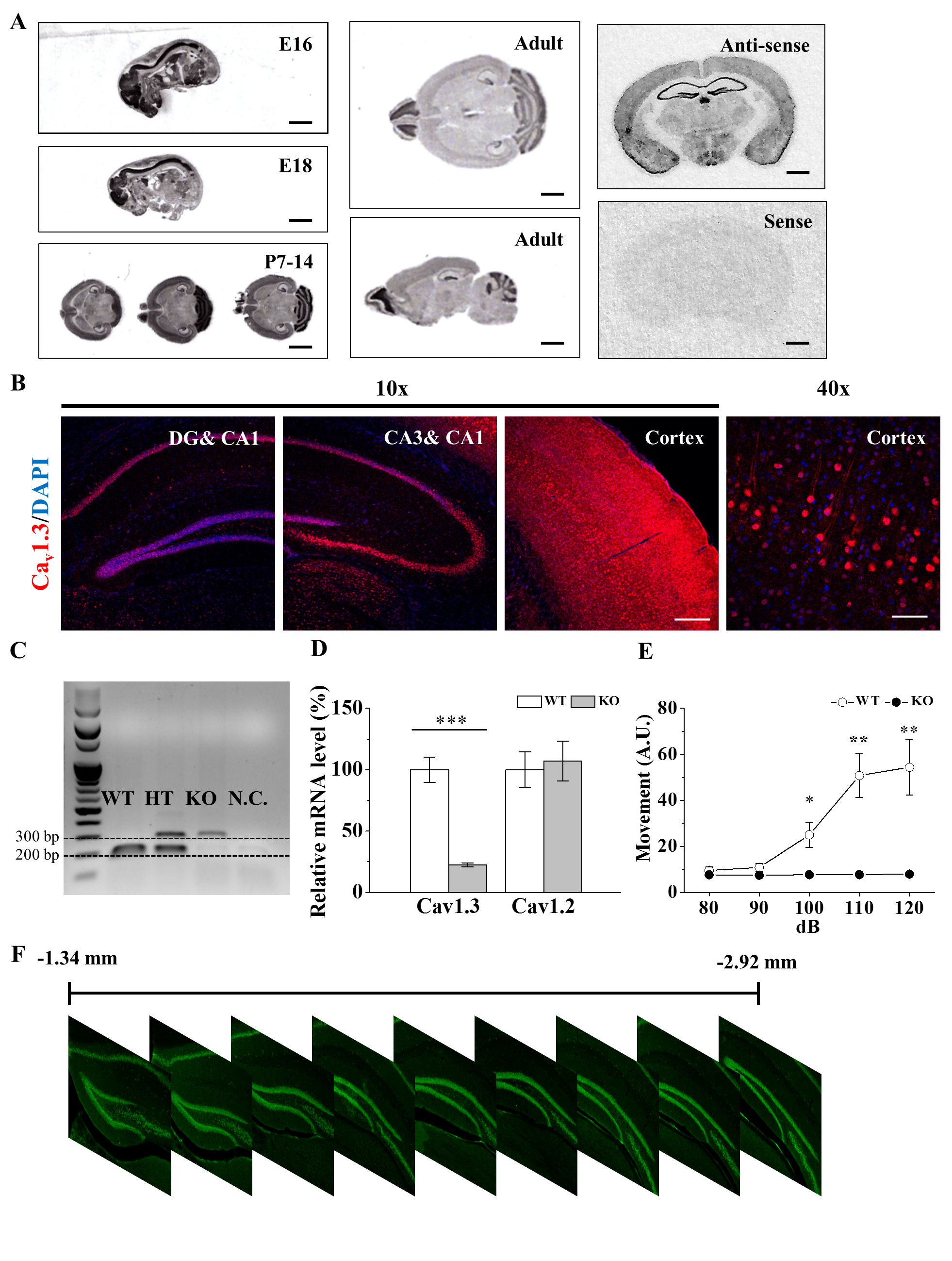

Supplement: S1 Fig — (TIF) [file pone.0181138.s001.tif]

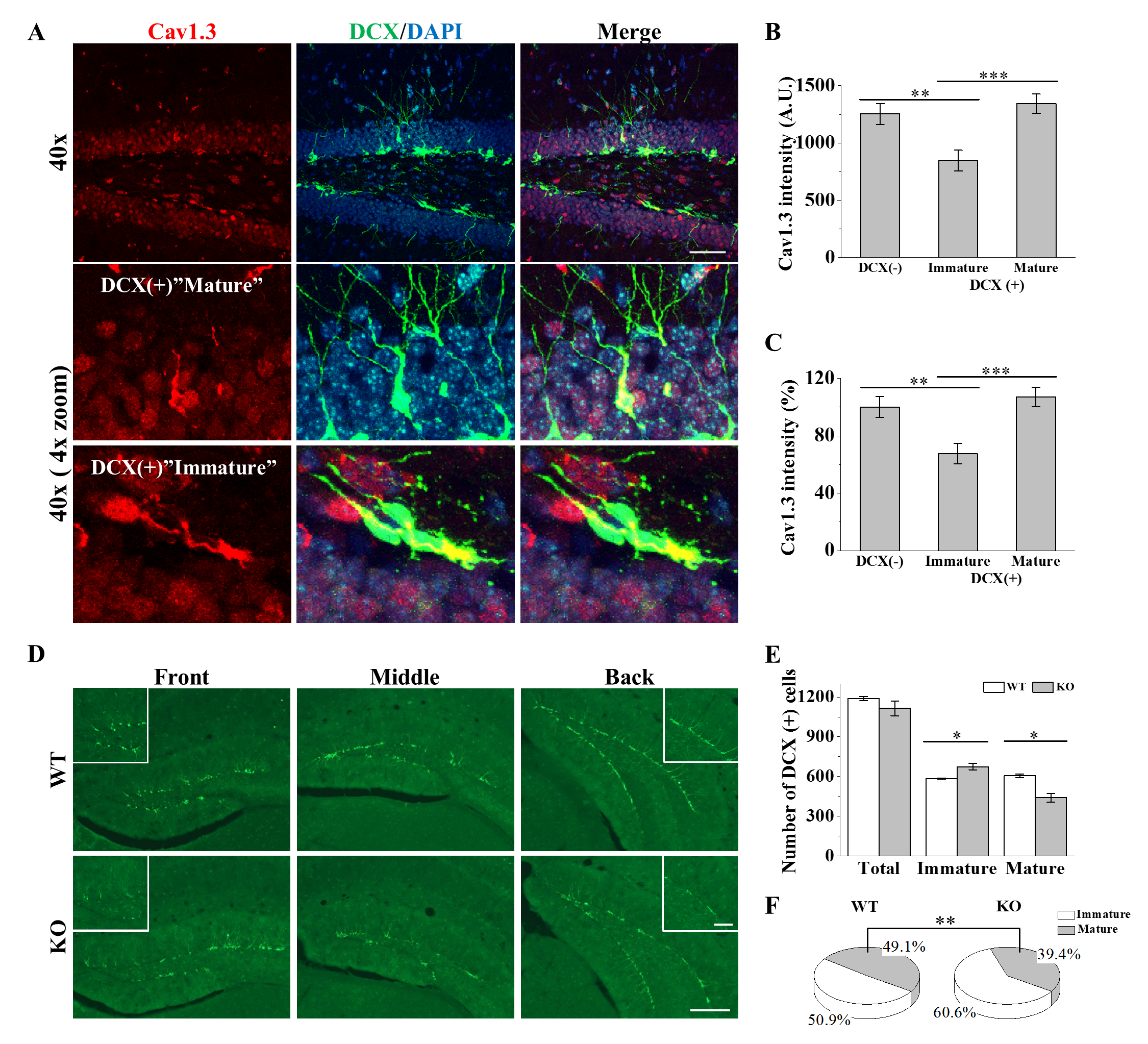

Supplement: S2 Fig — (TIF) [file pone.0181138.s002.tif]

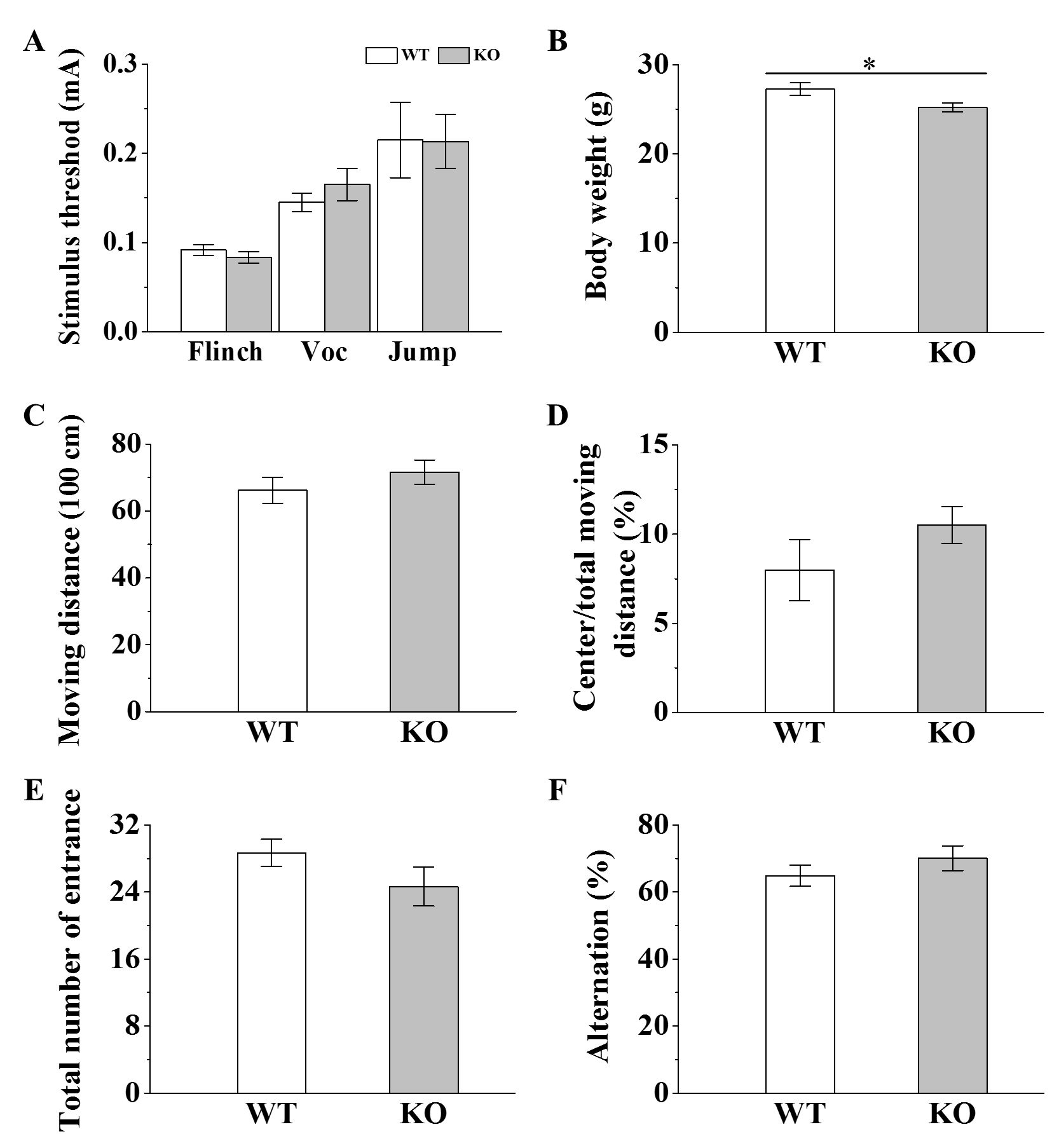

Supplement: S3 Fig — (TIF) [file pone.0181138.s003.tif]

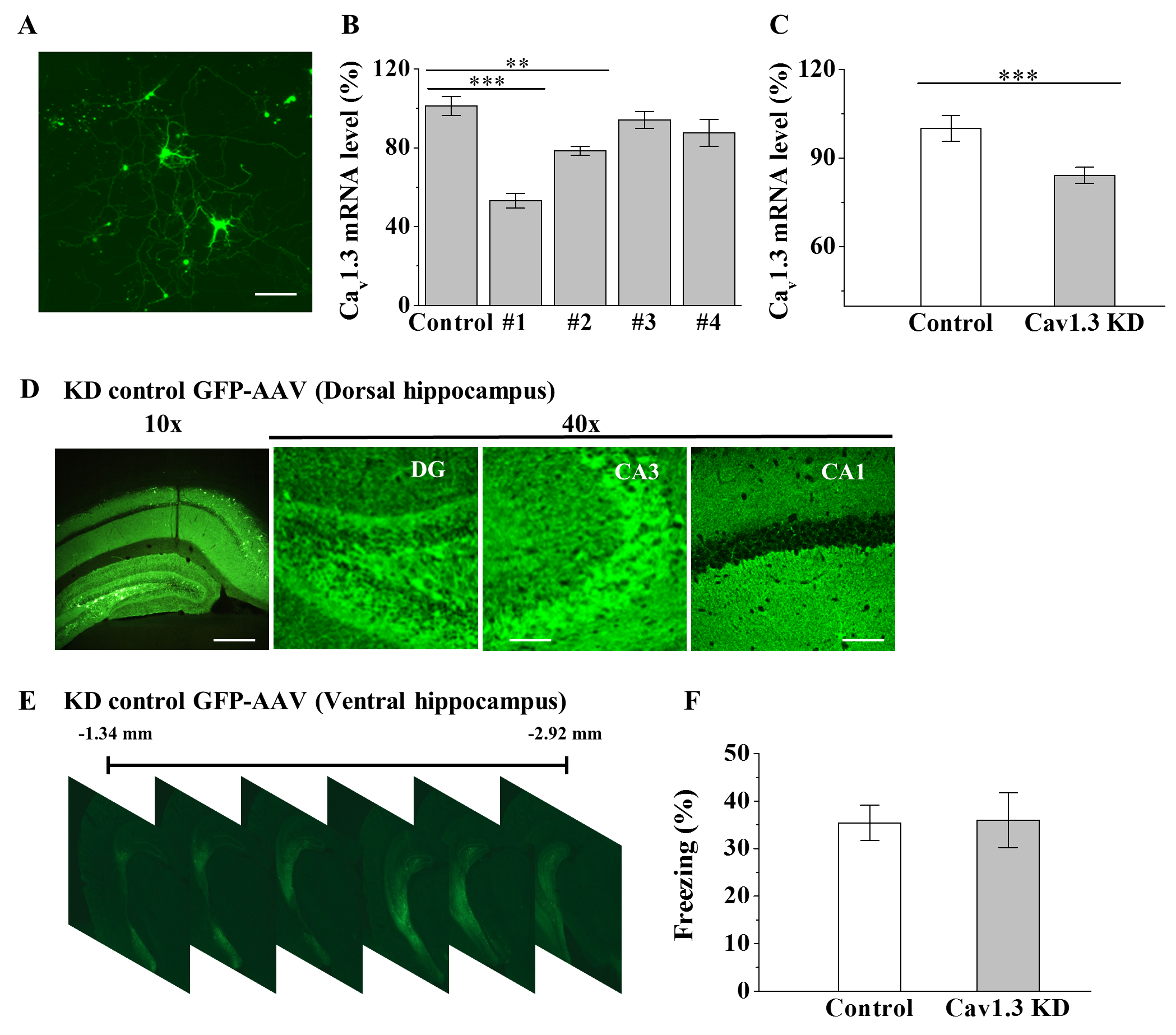

Supplement: S4 Fig — (TIF) [file pone.0181138.s004.tif]

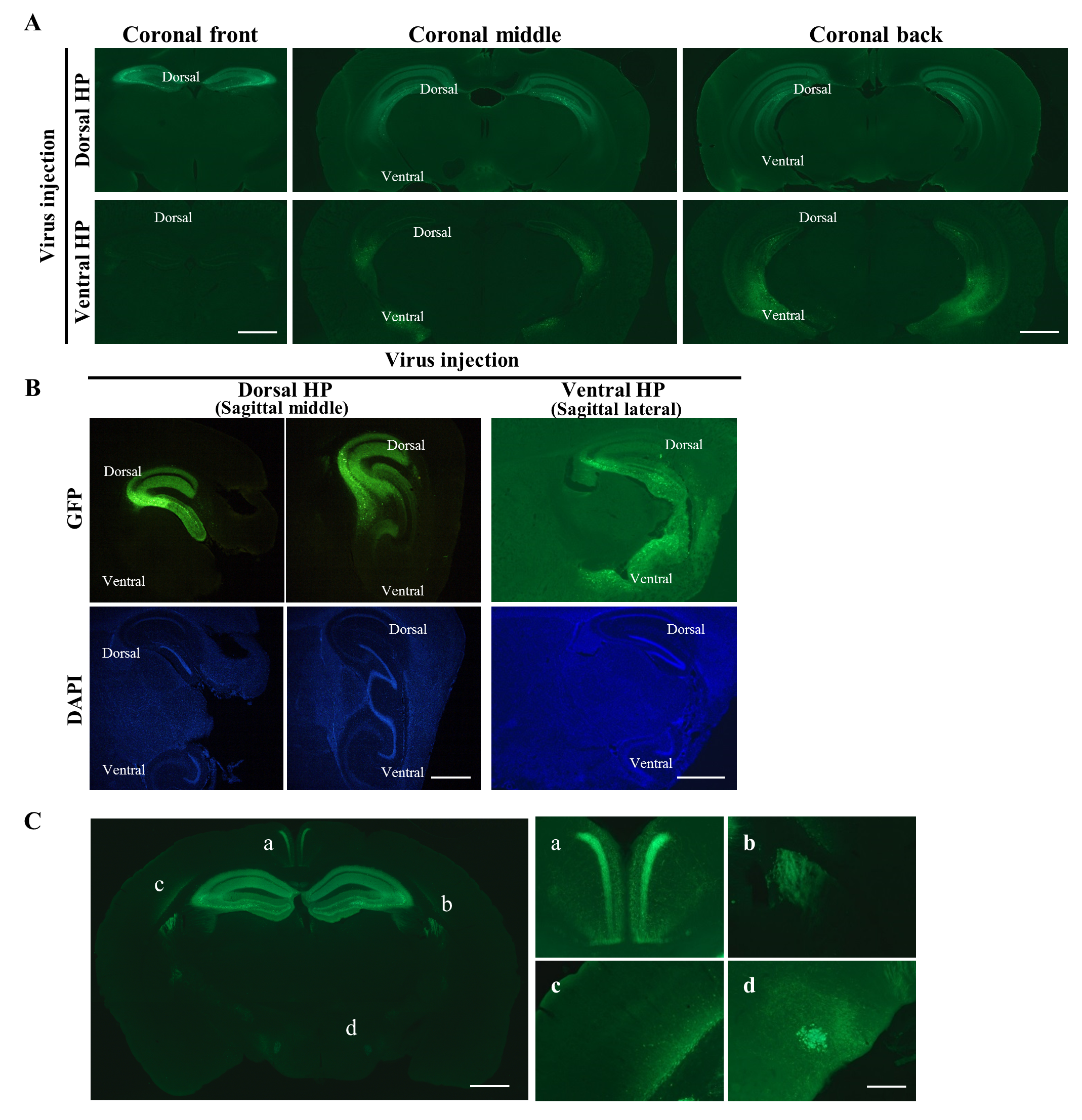

Supplement: S5 Fig — (TIF) [file pone.0181138.s005.tif]

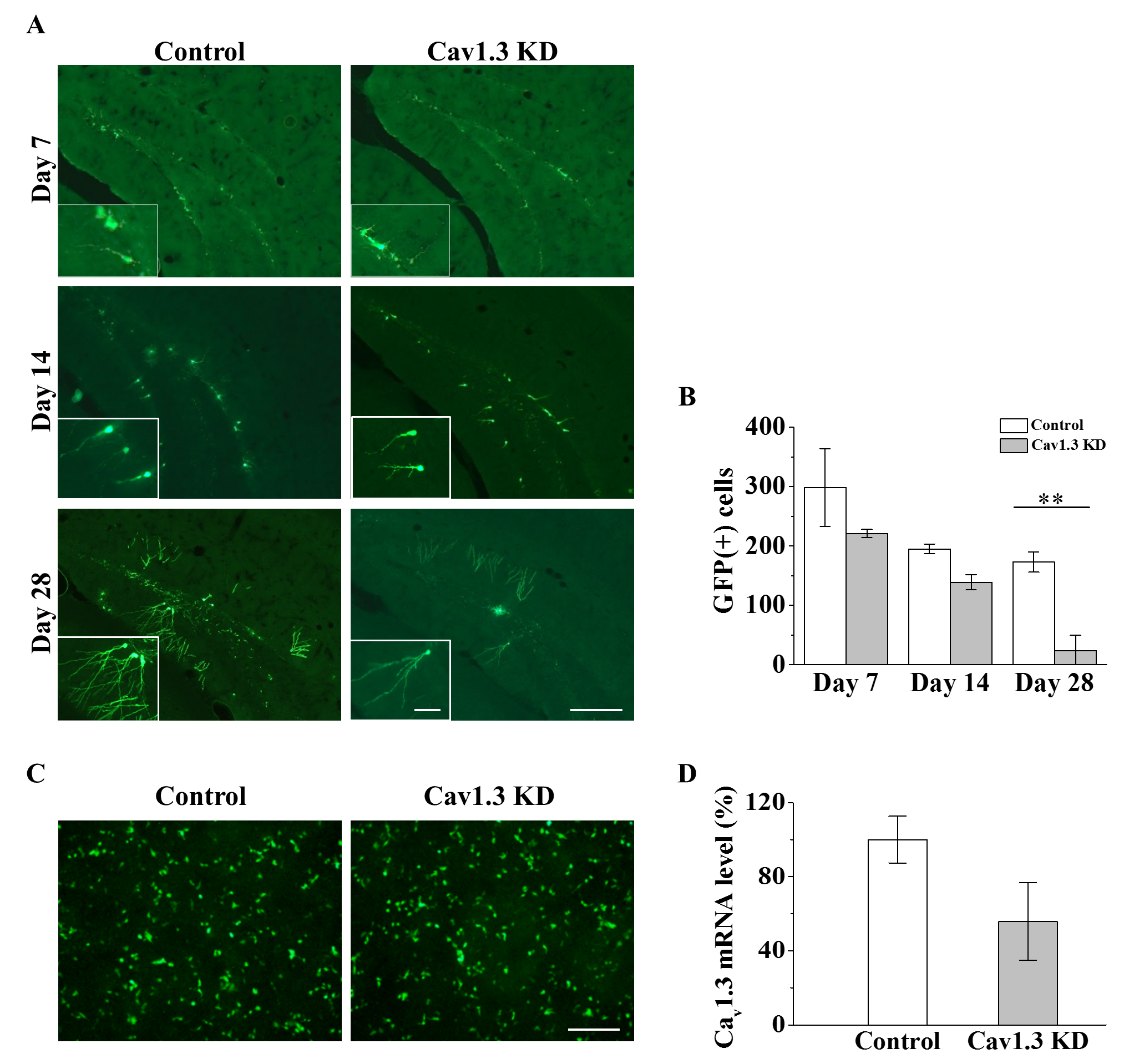

Supplement: S6 Fig — (TIF) [file pone.0181138.s006.tif]
