# Supplementary material for: Reduction of Cav1.3 channels in dorsal hippocampus impairs the development of dentate gyrus newborn neurons and hippocampal-dependent memory tasks
Source: PLoS One. 2017 Jul 17;12(7):e0181138. doi: 10.1371/journal.pone.0181138 (PMC5513490; doi:10.1371/journal.pone.0181138)
Supplement: S1 File — (DOCX) [file pone.0181138.s007.docx]

**Supporting Information**

**S1 Fig. Characterization of Ca_v_1.3 KO mice.**
(A) Representative images of *in situ* hybridization of Ca_v_1.3 mRNA during mouse development. Higher intensity of Ca_v_1.3 mRNA is shown as darker colors. E, embryonic day; P, postnatal day. *Scale bar*, 1 mm. (B) Representative images of immunohistochemistry of Ca_v_1.3 in mouse brain (8-week old). Low and high magnification images of dorsal hippocampus and cortex. *Scale bar*s, 200 µm (10x) and 50 µm (40x). (C) PCR Genotyping of Ca_v_1.3 KO (-/-), HT (+/-) and WT (+/+) mice. (D) Quantification of mRNA levels of Ca_v_1.3 and Ca_v_1.2 in the dorsal hippocampus of Ca_v_1.3 KO and WT mice. (Ca_v_1.3, WT, 100 ± 10.45%, n = 9, KO, 22.56 ± 1.61%, n = 6, p < 0.0001, Ca_v_1.2, WT, 100 ± 14.54%, n = 6, KO, 106.97 ± 16.24%, n = 6, *p* = 0.799, n = 3 animals per group). (E) Startle responses to auditory stimuli of Ca_v_1.3 KO and WT mice. (80 db, WT, 9.5 ± 1.63, KO, 7.63 ± 0.56, *p* = 0.31; 90 db, WT, 10.83 ± 1.80, KO, 7.42 ± 0.63, *p* = 0.115; 100 db, WT, 24.98 ± 5.56, KO, 7.68 ± 0.86, *p* = 0.015; 110 db, WT, 50.8 ± 9.47, KO, 7.72 ± 0.67, *p* = 0.002; 120 db, WT, 54.4 ± 12.11, KO, 7.92 ± 0.60, *p* = 0.005, n = 5 animals per group). Two-way ANOVA, F_G_ = 605.00, *p* = 0.000; F_S_ = 112.19, *p* = 0.000; F_T_ = 30.55, *p* = 0.000; F_G+S_ = 109.18, *p* = 0.000; F_G+T_ = 25.95, *p* = 0.000; F_S+T_ = 9.29, *p* = 0.000; F_G+S+T_ = 9.70; *p* = 0.000. (F) Representative images of 9 sections of dorsal hippocampus where BrdU (+) cells were quantified. Sequential images were collected from -1.34 to -2.92 mm to the bregma. Interval between sections was 200 µm. NeuN is shown in green.

**S2 Fig. Expression of Ca_v_1.3 and DCX of dorsal hippocampus in Ca_v_1.3 WT and KO mice.**(A) Representative images of expression of Ca_v_1.3 (red) and DCX (green) in Ca_v_1.3 WT mouse. DCX (+) “mature” cells indicates cells which express DCX and localize at granule cell layer of DG with highly branched dendrites. DCX (+) “immature” cells indicates cells which express DCX and localize at subgranular zone with little dendritic branches. DAPI is in blue. *Scale bar*s, 50 µm (40x) and 10 µm (40x/4x-zoom).
(B) Quantification of intensity of soma Ca_v_1.3 fluorescence from DCX (+) cells and DCX (-) cells shown in (A). A. U., arbitrary unit. (DCX (-), 1253.4 ± 90.6, n = 12; DCX (+) Immature, 847.01 ± 90.39, n = 12; DCX (+) Mature, 1342.64 ± 84.46, n = 12, p (DCX (-) - Immature) = 0.004, *p* (Immature - Mature) = 0.001).
(C) Normalized intensity of soma Ca_v_1.3 in DCX (+) cells and DCX (-) cells shown in (A). (DCX (-), 100 ± 7.23, DCX (+) Immature, 67.58 ± 7.21, DCX (+) Mature, 107.12 ± 6.74, p (DCX (-) - Immature) = 0.004, *p* (Immature - Mature) = 0.0001).
(D) Representative images of expression of DCX in the dorsal hippocampus of Ca_v_1.3 WT and KO mice. Images (front, middle, back) are from the 9 sections. *Scale bar*, 250µm and 100 µm (inset, 2.x).
(E) Quantification of DCX (+) cells in Ca_v_1.3 WT and KO mice. (Total, WT, 1189 ± 14.01, KO, 1113.67 ± 55.44; Immature, WT, 583.67 ± 3.18, KO, 674 ± 23.59, *p* = 0.019; Mature, WT, 605.33 ± 14.66, KO, 439.67 ± 33.65, *p* = 0.011; n = 3 per groups).
(F) Percentage of mature and immature of DCX (+) cells in Ca_v_1.3 WT and KO mice. (Immature, WT, 49.1 ± 0.66, KO, 60.62 ± 1.2, *p* = 0.001; Mature, WT, 50.9 ± 0.66%, KO, 39.38 ± 1.19%, *p* = 0.001; n = 3 animals per groups).

**S3 Fig. Neurological screening tests in Ca_v_1.3 KO mice.**(A) Threshold measurement of electric shocks against three categories of behavioral responses. Voc indicates vocal responses. (Flinch, WT, 0.092 ± 0.006 mA, n = 6; KO, 0.083 ± 0.007 mA, *p* = 0.375; Voc, WT, 0.15 ± 0.01 mA, n = 6, KO, 0.17 ± 0.02 mA, *p* = 0.357; Jump, WT, 0.22 ± 0.04 mA, n = 6, KO, 0.21 ± 0.03 mA, n = 6, *p* = 0.975). (B) Body weight measurement. (WT, 27.25 ± 0.72 g, n = 14; KO, 25.21 ± 0.50 g, n = 14, *p* = 0.028). (C-D) Open field tests. (C) Total moving distance. (WT, 6618.35 ± 384.86 cm, n = 6; KO, 7165.03 ± 362.09 cm, n = 6, *p* = 0.391). (D) Percentage of moving distance within the center area over the total moving distance. (WT, 7.94 ± 1.71%, n = 6; KO, 10.52 ± 1.04%, n = 6, *p* = 0.235). (E-F) Y-Maze test. (E) The number of entries. (WT, 28.67 ± 1.64, n = 10; KO, 24.67 ± 2.33, n = 10, *p* = 0.180). (F) Percentage of spontaneous alternation. (WT, 64.88 ± 3.15%, n = 10; KO, 70.08 ± 3.70%, n = 10, *p* = 0.300). * indicates *p* < 0.05.

**S4 Fig. Characterization of AAV-mediated Ca_v_1.3 KD *in vitro* and *in vivo*.**(A-B) GFP-AAV-Ca_v_1.3 KD efficiency in primary cortical neurons using qRT-PCR. (A) Representative image of primary cortical neurons at 24 hours after transfection with KD control GFP-AAV plasmid. *Scale bar*, 20 µm. (B) Ca_v_1.3 mRNA levels in primary cortical culture cells at 48 hours after transfection with a candidate AAV-Ca_v_1.3 KD or control plasmid using qRT-PCR. (Control, 101.30 ± 4.83%, Candidate #1, 53.08 ± 3.75%, Candidate #2, 78.39 ± 2.33%, Candidate #3, 94.14 ± 4.33%, Candidate #4, 87.57 ± 6.82%, *p* < 0.00001 (control-candidate #1), *p* < 0.00001 (control-candidate #2), n = 16 samples, n = 4 wells per group). One-way ANOVA, F = 21.54, *p* = 0.000. (C) AAV-Ca_v_1.3 KD efficiency at 2-week of viral infection in dorsal hippocampus using qRT-PCR. (Control, 100 ± 8.44%, n = 12; Ca_v_1.3 KD, 85.26 ± 2.97%, n = 12, n = 4 animals per group, *p* = 0.009). (D) Representative images of GFP expression at 2 weeks after KD control GFP-AAV injection into the dorsal hippocampus of F1 mouse. *Left*, sequential images of entire dorsal hippocampus infected with KD control GFP-AAV; *middle*, an example image of dorsal hippocampal sections showing the penetrating needle area; *right*, higher magnification images of CA1, CA3 and DG. *Scale bars*, 200 µm (10x) and 50 µm (40x). (E) Representative images of GFP expression at 2 weeks after KD control GFP-AAV injection into ventral hippocampal area of F1 mouse. *Scale bar*, 200µm (10 x). (F) Freezing responses of the recent CFC memory test of AAV-Ca_v_1.3 KD mouse in ventral hippocampal area. (Control, 35.43 ± 3.71%, n = 8; Ca_v_1.3 KD, 36.44 ± 5.77%, n = 8, *p* = 0.934).

**S5 Fig. Comparison of GFP expressions at dorsal and ventral hippocampus mediated by AAV-Ca_v_1.3 KD injections, respectively.**(A) Representative coronal section images of GFP expression when AAV-Ca_v_1.3 KD injection was made into dorsal (top) and ventral (bottom) hippocampus. In ventral hippocampal injection, there are little GFP signals in the dorsal sections. Scale bar, 1 mm (2.5x) and 250 µm (10x).
(B) Representative sagittal section images of GFP expression (top) when AAV-Ca_v_1.3 KD injection was made into dorsal (left) and ventral (right) hippocampus. Bottom images show the DAPI staining of the same top sections. In dorsal hippocampal viral injection, GFP signals are mostly confined in dorsal area of hippocampous. Scale bar, 1 mm (2.5x) and 1 mm (10x)
(C) Representative images of GFP (+) regions other than hippocampus when AAV-Ca_v_1.3 KD injection was made into the dorsal hippocampus. (a) Retrosplenial granular area. (b) Fimbria of the hippocampus. (c) Cortex. (d) Fornix. Scale bar, 500 µm (2.5x) and 250 µm (1x).

**S6 Fig. Characterization of retrovirus-mediated GFP (+) Ca_v_1.3 KD *in vitro* and *in vivo*.**(A) Representative images of GFP expression mediated by scrambled control virus (left) and retrovirus-Ca_v_1.3 KD (right) in dorsal hippocampus of F1 mouse at indicated days of post viral injection. *Scale bar*, 250 µm and 50 µm (insets).
(B) Quantification of GFP (+) cells infected with control and retrovirus-Ca_v_1.3 KD dorsal hippocampus. (Day 7, Control, 298.5 ± 65.5, n = 2, KD, 221 ± 7, n = 2; Day 14, Control, 195 ± 8.33, n = 3, KD, 139 ± 13, n = 3; Day 28, Control, 173 ± 16.96, n = 7, KD, 23. 71 ± 25.9, n = 8, *p* = 0.001)
(C) Representative images of expression of GFP for titer measurements by retrovirus-Ca_v_1.3 KD and control in HEK 293T cells at 36 hours after infection.
(D) Ca_v_1.3 mRNA levels in HT22 cells at 48 hours after transfections with retrovirus-Ca_v_1.3 KD or control plasmid using qRT-PCR. (Control, 100 ± 12.65%, n = 9; Cav1.3 KD, 55.85 ± 20.98%, n = 9, *p* = 0.090).

**Materials and Methods**

**In situ hybridization**
Procedure of in situ hybridization in this study was according to Xiao et al. (2007) [1]. To produce probe of specific Ca_v_1.3 mRNA, we first isolated total RNA from hippocampus of mice using Trizol reagent (Gene All, Korea) and the RNA was synthesized to CDNA by M-MLV reverse transcriptase (Mbiotech, Korea). Ca_v_1.3 specific probe was amplified by PCR with primers; sense, 5'-AGG CAA ACT ATG CAA GAG GCA CCA G-3' and antisense, 5'-ATT CCA TCC ATT CCT AAC GTA AGC-3' and subcloned into pCRⅡ-TOPO vector. Radioactive probes were generated and used visualization. The detailed procedure followed the previous study [2].

**Genotyping**To genotype Ca_v_1.3 KO mice, we extracted DNA from tails of 3 weeks old mice. Genotyping was done by PCR. Ca_v_1.3 KO mice were generated by insertion of neomycin resistance gene (neo) at exon 2 site. The primers were generated by Genotech (Korea): neo-sense: 5’-GCA AAC TAT GCA AGA GGC ACC AG-3’; neo-reverse: 5’- GGG AGA GAG ATC CTA CAG GTG G-3’; PGK22: 5’-CTG ACT AGG GGA GGA GTA GAA G-3’in PCR reaction. The sizes of PCR product in wild type allele and mutant allele are 180bp and 300bp, respectively [3].

**DCX staining**To identify the expression of Ca_v_1.3 in DCX (+) cells, the serial sections in the dorsal hippocampus of Ca_v_1.3 WT and KO mice were stained with goat anti-DCX antibody (1: 50, Santa cruze, USA; SC-8066) and rabbit anti-Ca_v_1.3 antibody (1: 200, Alomone Lab, Israel, #ACC-311). Blocking solution was 5% normal goat serum with 0.3% triton X-100 in PBS.

**Open field test**Open field test was done to determine the gross locomotor activity and exploration habits of mouse. The open field chamber was a white acrylic box (40 x 40 x 40 cm). Mouse was tested for 30 min in the box illuminated with indirect white light. At the beginning of the test, mouse was placed in the center with its head facing the wall. The total walking distance and the time spent in the center area were monitored and analyzed with Ethovision 3.1 (Noldus Information Technology, Leesburg, VA). After the test, mice were returned to its home cage and the box was thoroughly wiped using 70% EtOH and distilled water (DW).

**Acoustic startle response test**
Chamber for acoustic startle test (SR-LAB; San Diego Instruments, San Diego, Calif, USA) was ventilated and lighted. A mouse was placed in a startle enclosure and its startle responses were detected by the piezoelectric sensor hermetically sealed to the underside of the animal enclosure. The sensor recorded the pressure whenever the animal jumped. A high frequency loudspeaker inside the chamber produced both a continuous background noise as well as the various acoustic stimuli. Test session was consisted of a 10 min adaptation period followed by an assessment period of responses to auditory stimuli. Each mouse was presented 60 trials over 20 min test session, and five different sound levels (80, 90, 100, 110, 120 dB) with 40 ms duration were presented twelve times in a semi-random sequence with random intervals (1~40 s).

**Y-Maze**
A Y-maze consists of three arms of equal size. Each arm is a V-shape corridor made of black plastic, 32.5 cm long, 3 cm wide and 15 cm high. The area was cleaned using 70% EtOH and DW between trials to avoid odor trails. The Y-maze test was performed under 50 lux lighting conditions. Mouse began the single trial at the end of one arm and was allowed to freely explore the Y-maze for 8 min. Spontaneous alternation was measured. A correct alternation is when the mouse moved to the other two arms without retracing its former steps.

**Shock sensitivity test**
For detection of sensitivity to electrical shocks, mouse was placed inside an electrical shock test chamber (Med association, USA) and then exposed to electrical foot-shock of 0.05 mA for 1 s, which was increased by 0.01 mA step up to 0.5 mA with 30 s interval between shocks. Responses to shocks were categorized as flinch, vocalization and jump. Flinch is the response that mouse doesn’t move for a moment while four feet touching with grid after a foot-shock. Vocalization (Voc) is the sound when mouse shouts for several seconds after a foot-shock. Jump is the action when mouse lifted off four feet immediately after a foot-shock.

**ANOVA-Statistical test**

**Fig 1C**

| **Bonferroni** | | | | |
| --- | --- | --- | --- | --- |
| **Source** | | | ***p* value** | |
| **Day 3** | **Day 7** | | 0.716 | |
|  | **Day 14** | | 0.101 | |
|  | **Day 28** | | 0.000 | |
| **Day 7** | **Day 3** | | 0.716 | |
|  | **Day 14** | | 0.001 | |
|  | **Day 28** | | 0.000 | |
| **Day 14** | **Day 3** | | 0.101 | |
|  | **Day 7** | | 0.001 | |
|  | **Day 28** | | 0.045 | |
| **Day 28** | **Day 3** | | 0.000 | |
|  | **Day 7** | | 0.000 | |
|  | **Day 14** | | 0.045 | |
| **Source** | | **F** | | ***p* value** |
| **Genotype** | | 66.17 | | 0.000 |
| **Time** | | 15.22 | | 0.000 |
| **Genotype + Time** | | 3.20 | | 0.031 |

**Fig 1D**

| **Source** | **F** | | ***p* value** |
| --- | --- | --- | --- |
| **Within Groups** | 20.913 | | 0.000 |
| **Dunnett t** | | | |
| **Source** | | ***p* value** | |
| **Day 3 - Day 7** | | 0.993 | |
| **Day 3 - Day 14** | | 0.001 | |
| **Day 3 - Day 28** | | 0.000 | |

**Fig 1E**

| **Source** | | **F** | | ***p* value** |
| --- | --- | --- | --- | --- |
| **Within groups** | | 8.42 | | 0.001 |
| **Bonferroni** | | | | |
| **Source** | | | ***p* value** | |
| **DG** | **CA1** | | 0.158 | |
|  | **CA3** | | 0.001 | |
| **CA1** | **DG** | | 0.158 | |
|  | **CA3** | | 0.142 | |
| **CA3** | **DG** | | 0.001 | |
|  | **CA1** | | 0.142 | |

**Fig 2B**

| **Source** | **F** | ***p* value** |
| --- | --- | --- |
| **Genotype** | 3.80 | 0.061 |
| **Time** | 59.12 | 0.000 |
| **Genotype + Time** | 0.84 | 0.444 |

**Fig 2C**

| **Source** | **F** | ***p* value** |
| --- | --- | --- |
| **Genotype** | 4.61 | 0.040 |
| **Time** | 1.82 | 0.179 |
| **Genotype + Time** | 1.90 | 0.168 |

**Fig 3B**

| **Source** | **F** | ***p* value** |
| --- | --- | --- |
| **Genotype** | 26.96 | 0.000 |
| **Time** | 73.08 | 0.000 |
| **Genotype + Time** | 0.68 | 0.410 |

**Fig 3C**

| **Source** | **F** | ***p* value** |
| --- | --- | --- |
| **Genotype** | 1.96 | 0.162 |
| **Time** | 76.56 | 0.000 |
| **Genotype + Time** | 10.31 | 0.001 |

**Fig 3D-E**

| **Source** | **F** | | | ***p* value** |
| --- | --- | --- | --- | --- |
| **Genotype** | 10.54 | | | 0.001 |
| **Time** | 27.18 | | | 0.000 |
| **Distance** | 92.87 | | | 0.000 |
| **Genotype + Time** | 34.97 | | | 0.000 |
| **Genotype + Distance** | 1.23 | | | 0.270 |
| **Time + Distance** | 23.76 | | | 0.000 |
| **Genotype + Time + Distance** | 0.92 | | | 0.504 |
| **Repeated measure** | | | | |
| **Source : 20µm** | | **F** | ***p* value** | |
| **Time** | | 19.948 | 0.000 | |
| **Time + Genotype** | | 1.363 | 0.245 | |
| **Source : 40µm** | | **F** | ***p* value** | |
| **Time** | | 6.131 | 0.014 | |
| **Time + Genotype** | | 2.452 | 0.119 | |
| **Source : 60µm** | | **F** | ***p* value** | |
| **Time** | | 0.302 | 0.583 | |
| **Time + Genotype** | | 5.462 | 0.021 | |
| **Source : 80µm** | | **F** | ***p* value** | |
| **Time** | | 3.070 | 0.082 | |
| **Time + Genotype** | | 3.070 | 0.082 | |
| **Source : 100µm** | | **F** | ***p* value** | |
| **Time** | | 11.797 | 0.001 | |
| **Time + Genotype** | | 0.839 | 0.365 | |

**Fig 4B**

| **Source** | **F** | | ***p* value** | |
| --- | --- | --- | --- | --- |
| **Genotype** | 18.24 | | 0.000 | |
| **Time** | 17.88 | | 0.000 | |
| **Genotype + Time** | 2.31 | | 0.106 | |
| **Repeated measure** | | | | |
| **Source** | | **F** | | ***p* value** |
| **Time** | | **21.133** | | **0.000** |
| **Time + Genotype** | | **1.392** | | **0.277** |

**Fig 4D**

| **Source** | **F** | | ***p* value** | |
| --- | --- | --- | --- | --- |
| **Genotype** | 2.45 | | 0.120 | |
| **Time** | 17.47 | | 0.000 | |
| **Genotype + Time** | 2.33 | | 0.078 | |
| **Repeated measure** | | | | |
| **Source** | | **F** | | ***p* value** |
| **Time** | | 21.133 | | 0.000 |
| **Time + Genotype** | | 1.392 | | 0.277 |

**Fig 5C**

| **Source** | | **F** | | | ***p* value** | |
| --- | --- | --- | --- | --- | --- | --- |
| **Genotype** | | 15.09 | | | 0.000 | |
| **Time** | | 27.49 | | | 0.000 | |
| **Genotype + Time** | | 6.12 | | | 0.004 | |
| **Bonferroni** | | | | | | |
| **Source** | | | | ***p* value** | | |
| **Day 1** | **Day 2** | | | 0.000 | | |
|  | **Day 23** | | | 0.001 | | |
| **Day 2** | **Day 1** | | | 0.000 | | |
|  | **Day 23** | | | 0.016 | | |
| **Day 23** | **Day 1** | | | 0.001 | | |
|  | **Day 2** | | | 0.016 | | |
| **Repeated measure** | | | | | | |
| **Source** | | | **F** | | | ***p* value** |
| **Time** | | | 19.816 | | | 0.000 |
| **Time + Genotype** | | | 5.315 | | | 0.022 |

**Fig 5F**

| **Source** | **F** | | ***p* value** | |
| --- | --- | --- | --- | --- |
| **Genotype** | 4.33 | | 0.041 | |
| **Time** | 32.07 | | 0.000 | |
| **Genotype + Time** | 0.14 | | 0.866 | |
| **Repeated measure** | | | | |
| **Source** | | **F** | | ***p* value** |
| **Time** | | 28.107 | | 0.000 |
| **Time + Genotype** | | 0.021 | | 0.980 |

**S1 Fig E**

| **Source** | | **F** | | ***p* value** |
| --- | --- | --- | --- | --- |
| **Genotype** | | 605.00 | | 0.000 |
| **Sound dB** | | 112.19 | | 0.000 |
| **Trials** | | 30.55 | | 0.000 |
| **Genotype + Sound dB** | | 109.18 | | 0.000 |
| **Genotype + Trials** | | 25.95 | | 0.000 |
| **Sound dB + Trials** | | 9.29 | | 0.000 |
| **Genotype + Sound dB + Trials** | | 9.70 | | 0.000 |
| **Bonferroni** | | | | |
| **Source** | | | ***p* value** | |
| **80db** | **90db** | | 1.000 | |
|  | **100db** | | 0.000 | |
|  | **110db** | | 0.000 | |
|  | **120db** | | 0.000 | |
| **90db** | **80db** | | 1.000 | |
|  | **100db** | | 0.000 | |
|  | **110db** | | 0.000 | |
|  | **120db** | | 0.000 | |
| **100db** | **80db** | | 0.000 | |
|  | **90db** | | 0.000 | |
|  | **110db** | | 0.000 | |
|  | **120db** | | 0.000 | |
| **110db** | **80db** | | 0.000 | |
|  | **90db** | | 0.000 | |
|  | **100db** | | 0.000 | |
|  | **120db** | | 1.000 | |
| **120db** | **80db** | | 0.000 | |
|  | **90db** | | 0.000 | |
|  | **100db** | | 0.000 | |
|  | **110db** | | 1.000 | |

**S4 Fig B**

| **Source** | | **F** | | ***p* value** |
| --- | --- | --- | --- | --- |
| **Within groups** | | 21.54 | | 0.000 |
| **Dunnett** | | | | |
| **Source** | | | ***p* value** | |
| **#1** | **Control** | | 0.000 | |
| **#2** | **Control** | | 0.001 | |
| **#3** | **Control** | | 0.530 | |
| **#4** | **Control** | | 0.061 | |

**Reference**

1. Xiao H, Chen X, Steele EC, Jr. Abundant L-type calcium channel Ca(v)1.3 (alpha1D) subunit mRNA is detected in rod photoreceptors of the mouse retina via in situ hybridization. Mol Vis. 2007;13:764-71. Epub 2007/06/15. PubMed PMID: 17563731; PubMed Central PMCID: PMCPMC2768761.

2. Chen C-C, Wada K, Jarvis ED. Radioactive in situ Hybridization for Detecting Diverse Gene Expression Patterns in Tissue. Journal of Visualized Experiments : JoVE. 2012;(62):3764. doi: 10.3791/3764. PubMed PMID: PMC3466668.

3. Platzer J, Engel J, Schrott-Fischer A, Stephan K, Bova S, Chen H, et al. Congenital deafness and sinoatrial node dysfunction in mice lacking class D L-type Ca2+ channels. Cell. 2000;102(1):89-97. PubMed PMID: 10929716.

**Statement of No Competing Interest**

Authors declare no competing interests.

*The authors have declared that no competing interests exist.*

**Acknowledgements**

We thank Drs. R. J. DiLeone, H. S. Shin, H. Song and F. H. Gage for their plasmids. We also thank D.-H. Choi, S. Song, and J. Lee for assisting statistical analysis and data quantification. This work was supported by the Brain Research Program through the National Research Foundation of Korea (NRF) funded by the Ministry of Science, ICT & Future Planning (http://www.nrf.re.kr/index) to C.-H.K. (Project No. 2016M3C7A1905119 and 2015M3C7A1 028392) and by the KIST Institutional Program (<https://www.kist.re.kr/kist_web/main/>) to C.-H.K. (Project No. 2E26820). *The funders had no role in study design, data collection and analysis, decision to publish, or preparation of the manuscript***.**
